# Comparative effectiveness of bone-protective interventions for aromatase inhibitors-induced bone loss in postmenopausal women with early breast cancer: a network meta-analysis

**DOI:** 10.3389/fonc.2025.1638370

**Published:** 2026-01-06

**Authors:** Yonghui Xu, Junjie Lai, Minjun Mai, Yi Tang, Zhouhan Wu

**Affiliations:** 1 The Graduate School, Guangxi University of Chinese Medicine, Nanning, China; 2 The Graduate School, Zhejiang Chinese Medical University, Hangzhou, China

**Keywords:** aromatase inhibitors, bone loss, bone-protective interventions, breast cancer, network meta-analysis

## Abstract

**Objectives:**

This network meta-analysis (NMA) aims to compare the efficacy of bone-protective interventions in preventing AIs-induced bone loss at lumbar spine and hip sites.

**Methods:**

We systematically searched PubMed, Embase, Cochrane Library, Web of Science, and clinical trial registries from inception to February 28, 2025, to identify randomized controlled trials (RCTs) evaluating bone-protective interventions in postmenopausal women with breast cancer receiving AI therapy. Primary outcomes were changes in bone mineral density (BMD) at lumbar spine and total hip at 12 and 24 months. Network meta-analysis was performed using Bayesian methods with R software and WinBUGS. Treatment rankings were assessed using surface under the cumulative ranking curve (SUCRA) values.

**Results:**

Seventeen RCTs involving 6,932 postmenopausal patients were included, comparing six interventions: denosumab, alendronate, zoledronic acid, ibandronate, risedronate, and eldecalcitol plus risedronate. At 12 months, denosumab demonstrated superior lumbar spine BMD improvement (SUCRA = 0.88, WMD = 5.63, 95% CI: 4.67-6.59), while ibandronate showed optimal hip BMD preservation (SUCRA = 0.94, WMD = 4.23, 95% CI: 2.91-5.43). At 24 months, denosumab maintained its advantage for lumbar spine (SUCRA = 0.83, WMD = 7.96, 95% CI: 5.38–10.52), whereas eldecalcitol plus risedronate showed the highest SUCRA ranking for hip preservation (SUCRA = 0.83, WMD = 6.38, 95% CI: 3.38–9.61). All active interventions significantly outperformed calcium plus vitamin D supplementation alone. Given that the eldecalcitol plus risedronate regimen was evaluated in only one randomized trial with a relatively small sample size, this finding should be interpreted cautiously.

**Conclusion:**

Among single agents, denosumab provides superior lumbar spine bone protection while ibandronate offers optimal hip bone preservation in AI-treated breast cancer patients. For extended therapy, combination regimens incorporating eldecalcitol with risedronate may show promising hip bone protection, though this conclusion is based on a single small-sample study and requires further validation. These findings support site-specific and duration-dependent treatment selection strategies. Further head-to-head trials are warranted to validate optimal treatment sequences.

**Clinical trial number:**

PROSPERO (CRD420251008444).

## 1 Introduction

Breast cancer (BC) is a leading cause of cancer-related death among women aged 20 to 50. with an annual incidence rate steadily increasing over the past few decades. Despite advances in screening and therapeutic interventions resulting in declining mortality rates (1). The extended survival duration has led to emerging health-related complications from protracted treatments, necessitating comprehensive management strategies (2–8). Nearly 80% of early breast cancer (EBC) cases in postmenopausal women are hormone receptor positive. for which a 5-year adjuvant treatment with aromatase inhibitors (AIs) remains the gold standard therapy (9, 10). However, AIs deplete serum estrogen, leading to a marked increase in bone resorption and significant decrease in bone mineral density (BMD) (11–13).

Previous meta-analyses have reported that anti-resorptive agents (zoledronate, ibandronate, risedronate, and denosumab) exhibit varying degrees of protective effects against BMD loss at the lumbar spine and total hip in AI-treated EBC patients (14, 15).

Despite these studies, there remains a paucity of clear comparative analyses regarding the efficacy differences among individual anti-resorptive agents. Furthermore, the unique characteristics of AI-treated EBC patients limit the categories of anti-resorptive drugs available for this population, with large-scale randomized controlled trials (RCTs) evaluating a broader range of agents being scarce. In this study, a systematic review and network meta-analysis (NMA) of both recent and historical evidence was performed for AI-treated EBC patients, aiming to directly and indirectly compare and rank the relative efficacy of different anti-resorptive agents in mitigating bone loss, and to provide evidence-based guidance for the selection of optimal bone-protective strategies in clinical practice.

## 2 Methods

This study followed the Preferred Reporting Items for Systematic Reviews and Meta-Analyses for Network Meta-Analyses (PRISMA-NMA) (39). This study protocol was registered at PROSPERO under the number CRD420251008444.

### 2.1 Literature search

To identify eligible studies, we conducted comprehensive systematic search in PubMed, Embase, Scopus, the Cochrane Library, and Web of Science from their inception until February 28, 2024. The search strategy incorporated MeSH terms and keywords related to AIs, bone loss, antiresorptive, and EBC (see Appendix for the detailed search strategy). Additionally, we manually screened the reference lists of all included studies and relevant review articles. We also evaluated any additional studies brought to our attention by experts in the field.

### 2.2 Inclusion and exclusion criteria

Population, Interventions, Comparisons, Outcomes and Study design (PICOS) (40) as a framework to formulate eligibility criteria of the included studies in our NMA were carefully designed as follows:

Inclusion criteria for studies: (1) Population: Post-menopausal women with EBC who are receiving aromatase inhibitor (AI) treatment. Moreover, the eligibility criteria regarding age and menopausal status of participants must be reported clearly in the original study. (2) Intervention: Patients in the intervention group received anti-bone-resorbing drugs combined with AIs treatment including a single anti-bone-resorbing drug, a combination of two or more anti-bone-resorbing drugs, or an anti-bone-resorbing drug plus other active drugs. (3) Comparison: Different anti-bone-resorbing medications from the treatment group, blank controls, placebo controls, or delayed-intervention controls. (4) Outcome: Changes in bone mineral density (BMD) of the lumbar spine and total hip measured as the percentage change relative to baseline or in a data format that allows for calculation. (5) Study design: The included articles were limited to randomized controlled trials (RCTs).

The exclusion criteria were as follows: (1) Studies involving non-postmenopausal women or patients with advanced BC. (2) Non-randomized controlled trials (such as observational studies). (3) Studies with incomplete or unextractable data. (4) Studies with a follow-up period of less than one year.

### 2.3 Study selection and data extraction

The retrieval records obtained from databases were imported into Endnote20 (Developed by Clarivate Analytics) for study selection by two researchers. After removing duplicates, two investigators independently screened titles/abstracts, with full-text review for final inclusion. Eligible studies’ references were hand-searched for additional RCTs. Disagreements were resolved by a senior researcher. Two investigators independently used Microsoft Excel (Microsoft, Redmond, Washington, USA) to extract data from the included RCTs. The extracted information and data encompassed five primary categories, containing basic study details (first author, publication year, and Trial acronym), participant information (sample size, average age, BMI), intervention details (AIs, anti - bone - resorbing drugs, and adjuvant medications): For bisphosphonates (zoledronic acid, ibandronate, risedronate, alendronate), different formulations (oral versus intravenous) were pooled and analyzed at the drug level rather than by formulation. This decision was made because only a few trials directly compared formulations, and separating them would have resulted in a sparse network with low statistical power. This strategy is consistent with previous meta-analyses in this field and is supported by the shared core mechanism of action of bisphosphonates across formulations, despite known differences in bioavailability and dosing frequency. All data entered in Excel were double-checked and verified by a third researcher to ensure consistency and validity. When only charts were provided in the literature, we used the Engauge Digitizer software (developed by Mark Mitchell’s company) to measure the pixel length of the coordinate axes for calibration. Subsequently, we obtained the coordinates or lengths of the required data points from the relevant coordinate axes. If there were crucial data missing, we would contact the corresponding author via email to obtain the necessary information.

### 2.4 Statistical analysis

Prior to conducting the network meta-analysis (NMA), the transitivity assumption was evaluated to ensure sufficient similarity among studies of different intervention groups. A Bayesian framework was employed for the NMA using R software (R Foundation), with the GeMTC and BUGSnet packages for analysis and ggplot2 for visualization (41, 42). The data were converted into a relative format and then input into the GeMTC package for analysis. For the primary outcomes (percentage change in BMD), weighted mean differences (WMDs) with 95% confidence intervals (CIs) were used as the effect measure. Posterior distributions were obtained using Markov chain Monte Carlo (MCMC) methods with 50,000 iterations after a burn-in of 20,000 iterations across four chains, with a thinning interval of 10 to reduce autocorrelation. Network plots were generated to illustrate the comparative relationships among various interventions, with each intervention represented by a node (size scaled proportionally to sample size) and connecting lines between nodes indicating direct comparisons (thickness corresponding to the number of available direct comparisons). Quantitative analyses included league tables showing WMDs with 95% CIs for all pairwise comparisons and forest plots reporting comparative results between intervention pairs. For intervention ranking, a hierarchy of competing interventions was established using the surface under the cumulative ranking curve (SUCRA), with values expressed as percentages (higher values indicating greater probability of being the optimal intervention choice).

### 2.5 Risk of bias within individual studies

The methodological quality of each RCT included in the study was independently assessed by two raters using the Cochrane Collaboration’s risk of bias tool (version 2.0, RoB 2).14 The risk of bias for each included RCT was evaluated based on five critical factors: the randomization process, deviations from the intended interventions, missing outcome data, outcome measurements, and choice of reported outcomes. Each of these domains was classified as having a low, unclear, or high risk of bias. In cases of disagreement between the two raters, a referee was involved to mediate and resolve the dispute.

### 2.6 Assessment of inconsistency and risk of bias across studies

The I² statistic was used to quantify heterogeneity among the results of each study, with I² > 50% indicating significant heterogeneity. The I² was assessed for both the overall network meta-analysis and individual study categories. For instances where the overall I² of the network meta-analysis exceeded 50%, network meta-regression was performed to investigate potential sources of heterogeneity. For the assessment of publication bias, funnel plots were examined when 10 or more studies reported the primary outcome. Additionally, the impact of significant publication bias on the meta-analysis results was evaluated using Egger’s test, with a p-value < 0.05 indicating significant bias.

### 2.7 Assessment of quality of evidence

The quality of evidence was assessed using Confidence in Network Meta-Analysis (CINeMA) (43, 44). This approach is based on a methodological framework evaluating six domains: within-study bias, reporting bias, indirectness, imprecision, heterogeneity, and incoherence. For each direct comparison, the confidence in evidence was rated as high, moderate, low, or very low based on the assessment of these six domains using the CINeMA web application (https://cinema.ispm.unibe.ch/).

## 3 Results

### 3.1 Literature search and study selection

**Figure 1:**
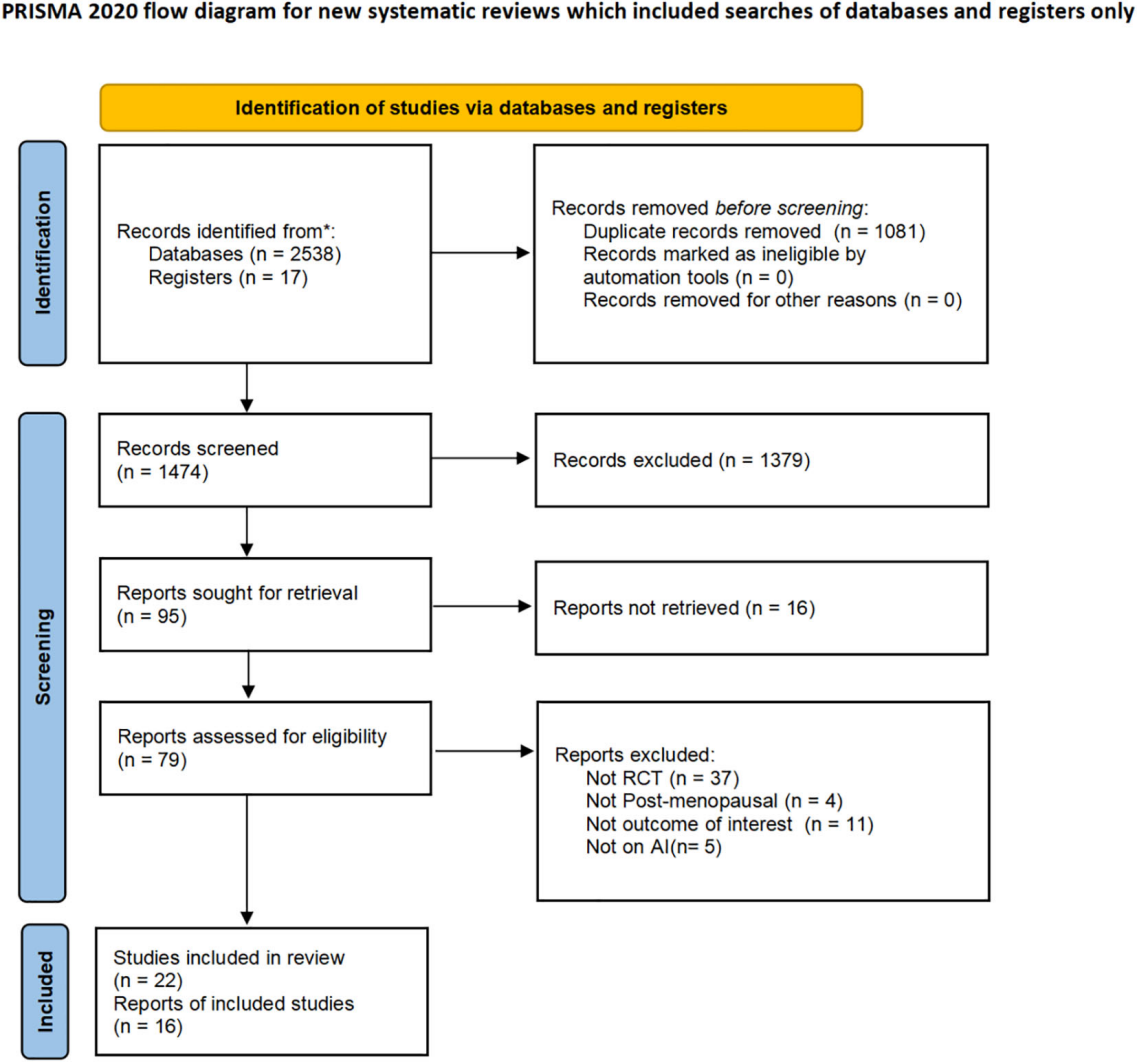
Preferred reporting items for systematic reviews and meta-analyses (PRISMA) 2020 flow diagram for systematic reviews.

Detailed information regarding the systematic literature search is presented in the PRISMA flow diagram (**Figure 1**). Through comprehensive searches across four databases, 2,538 potentially relevant articles were retrieved, with an additional 17 records identified through trial registries, totaling 2,555. After removing 1,081 duplicates, 1,474 unique records underwent title and abstract screening. Of these, 1,378 were excluded during initial assessment, leaving 96 articles for full-text retrieval. 16 articles were excluded due to inaccessible full texts, and 80 underwent full-text evaluation. During the full-text assessment phase, 57 articles were excluded for the following reasons: 37 non-randomized controlled trials, 11 without reporting target outcome measures, 5 not including patients receiving aromatase inhibitors therapy, and 4 involving non-postmenopausal populations. During the full-text assessment process, three studies require special explanation: the study by Lee et al. (45) was excluded due to inadequate randomization methodology, and the studies by Monda et al. (46) were excluded because they lacked extractable data required for the network meta-analysis.

Ultimately, 17 studies with a total of 23 articles were included in this network meta-analysis (16–37).

### 3.2 Characteristics of included studies

This meta-analysis incorporated 17 randomized controlled trials (RCTs) published between 2007 and 2025, conducted across multiple regions including North America, Europe, Asia, and Australia (**Table 1**). Cumulatively, 6,932 postmenopausal hormone receptor-positive (HR+) early breast cancer patients receiving adjuvant aromatase inhibitors (AIs) therapy were analyzed. Baseline characteristics across intervention groups demonstrated good between-group balance: patient age ranged from 56.0 to 68.0 years (overall median age 59.8 years), and the study with the largest sample size had 3,420 patients. Bone-protective interventions included zoledronic acid, ibandronate, risedronate, denosumab, and eldecalcitol combined with risedronate. Most subjects received standardized calcium (1000–1200 mg/d) and vitamin D (400–800 IU/d) supplementation as baseline therapy.

**Table 1:** Characteristics of the included studies.

Author trial acronym	Year	Mean age	Mean BMI	Aromatase inhibitor	Simple size	Intervention	Comparator	Duration of intervention	Co-intervention
Brufsky (16–18) Z-FAST	2007 2009 2012	60	29.3	Letrozole 2.5 mg daily	602	Zoledronic 4mg q6 months	Delayed Zol 4 mg q6 months if T- score decreased to ≤-2 or fracture	5 years	Ca 1000-1200 mg/d VitD 400-800 IU/d
Bundred (19) Editmann (20) Coleman (21) ZO-FAST	2008 2010 2013	58	27.5	Letrozole 2.5 mg daily	1065	Zoledronic 4mg q6 months	Delayed Zol 4 mg q6 months if T- score decreased to ≤-2 or fracture	5 years	Ca 500 mg/d VitD 400-800 IU/d
Hines (22) Wagner-Johnston (23) NO3CC	2009 2015	59	NA	Letrozole 2.5 mg daily	558	Zoledronic 4mg q6 months	Delayed Zol 4 mg q6 months if T- score decreased to ≤-2 or fracture	5 years	Ca 1000 mg/d VitD 400 IU/d
Llombart (24) E-ZO-FAST	2012	58	27.1	Letrozole 2.5 mg daily	527	Zoledronic 4mg q6 months	Delayed Zol4 mg q6 months ifT- score decreased to ≤-2 or fracture	12 months	Ca 500 mg/d VitD 400-800 IU/d
Takahashi (25)	2012	61	23.7	Letrozole 2.5 mg daily	194	Zoledronic 4mg q6 months	Delayed Zol4 mg q6 months ifT- score decreased to ≤-2 or fracture	12 months	NA
Sun (26)	2016	57	NA	Letrozole 2.5 mg daily	120	Zoledronic 4mg q6 months	Control	12 months	Ca 1000 mg/d VitD 400 IU/d
Lester (27, 28) ARIBON	2008 2012	68	27.5	Anastrozole 1 mg daily	50	Ibandronate 150 mg/month	Placebo	5 years	Ca 500 mg/d VitD 400 IU/d
Livia (29)	2019	61	NA	Exemestane or anastrozole	171	Ibandronate 150 mg every 28 days	Placebo	2 years	Ca 500 mg/d
Van Poznak (30) SABRE	2010	64	27.7	Anastrozole 1 mg daily	154	Risedronate 35 mg/week	Placebo	2 years	Ca 1000 mg/d VitD 400 IU/d
Markopoulos (31) ARBIb	2010	63	27.6	Anastrozole 1 mg daily	70	Risedronate 35 mg/week	Control	2 years	Ca 1000 mg/d Vit D400 IU/d
Greenspan (32)	2015	65	31	Anastrozole, letrozole, or exemestane	109	Risedronate 35 mg/week	Placebo	2 years	NA
Kadoya (33)	2016	NA	NA	NA	103	Risedronate 17.5 mg/week	Control	12 months	NA
Sestak (34)	2019	61	26.2	Anastrozole	260	Risedronate 35 mg/week	Placebo	5 years	NA
Ellis (35)	2008	59	27.8	Anastrozole, letrozole, or exemestane	252	Denosumab 60 mg subcutaneously every 6 months	Placebo	2 years	Ca 1000 mg/d VitD 400 IU/d
Gnant (36) ABCSG-18	2015	64	NA	Non-steroidal aromatase inhibitors	3420	Denosumab 60 mg subcutaneously every 6 months	Placebo	3 years	Ca 500 mg/d VitD 400 IU/d
Imanishi (37)	2025	65	22.5	Anastrozole, letrozole, or exemestane	196	Eldecalcitol 0.75μg/day and Risedronate 17.5 mg/week	Risedronate 17.5 mg/week	2 years	NA
Lomax (38)	2013	64	29	Anastrozole	146	alendronate (70mg)/week	Placebo	3 years	Ca 500 mg/day and Vitamin D 400 IU daily

Ca: Calcium; q: every; NA: Not Available; VitD: Vitamin D.

### 3.3 Risk of bias assessment

**Figure 2:**
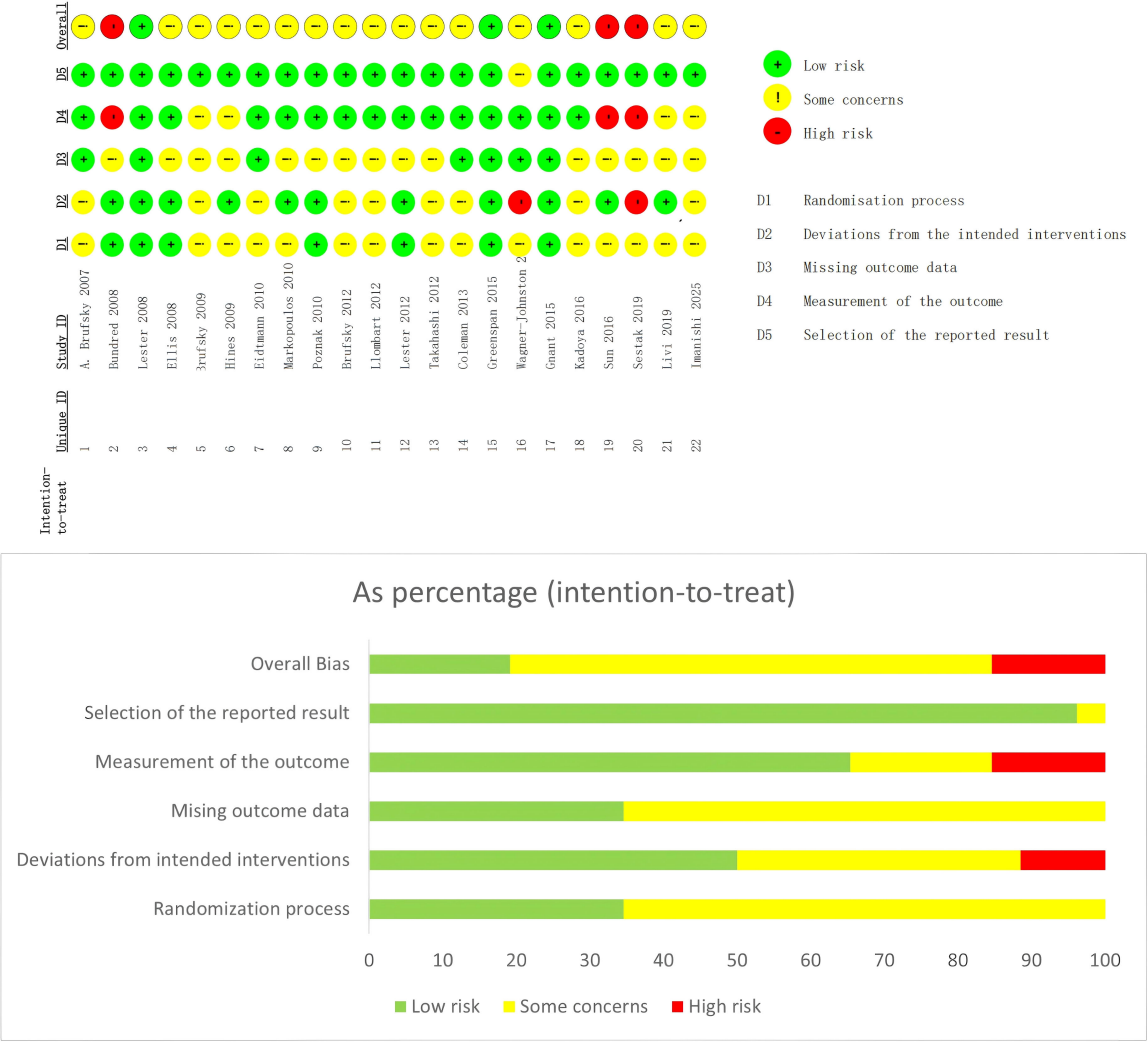
ROB2 risk of bias assessment.

All included studies reported adequate randomization methods and were rated as low risk. However, multiple studies did not implement adequate blinding procedures for investigators and participants, resulting in high-risk ratings in the deviations from intended interventions domain. Additionally, three studies were rated as high risk in the measurement of outcomes domain, indicating issues with outcome assessment procedures or inadequate reporting of methodological details. A detailed assessment of bias across all domains can be found on **Figure 2**.

### 3.4 Network structure and characteristics

**Figure 3:**
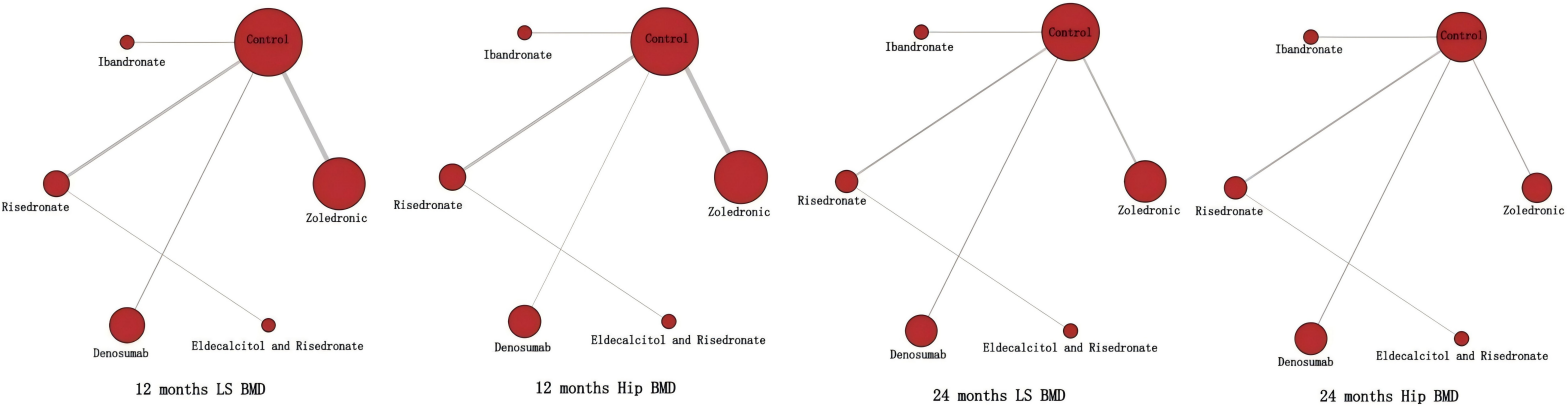
Network plots of interventions across time points and anatomical sites, with dot size proportional to sample size.

The evidence network exhibited a star-shaped topology, with all interventions being compared indirectly through a common control group (placebo or delay drug) (**Figure 3**). The network encompassed six interventions: zoledronic acid, ibandronate, alendronate, risedronate, denosumab, and eldecalcitol combined with risedronate. Indirect comparisons were formed only through the common control group (placebo or delay drug), with no direct head-to-head comparison evidence between active interventions. The network maintained identical connection patterns across four assessment time points (lumbar spine at 12 months, total hip at 12 months, lumbar spine at 24 months, and total hip at 24 months). Node sizes were proportional to the cumulative sample size for each intervention: the control group node was largest, followed by denosumab (n=1,876) and zoledronic acid (n=2,145), with alendronate (n=146) having the smallest node.

### 3.5 Network meta-analysis results

#### 3.5.1 Lumbar spine bone mineral density changes

**Figure 4:**
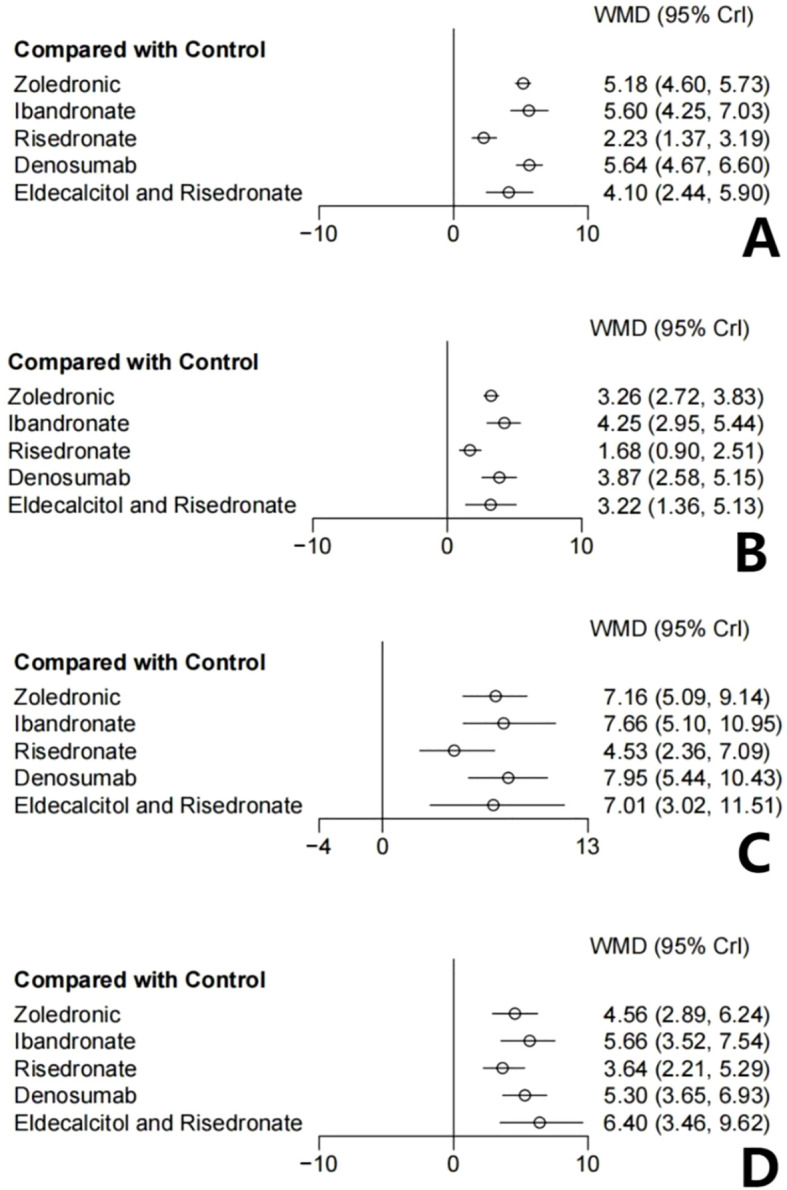
Forest plots for different time points and anatomical sites. **(A)** 12-month lumbar spine; **(B)** 12-month total hip; **(C)** 24-month lumbar spine; **(D)** 24-month total hip. CrI, credible interval; WMD, weighted mean difference; control group, placebo or delayed intervention.

At 12-month follow-up (**Figure 4A**, 16 studies, n=4,938), all interventions demonstrated superiority over the control group: denosumab (WMD = 5.63, 95% CI: 4.67-6.59), ibandronate (WMD = 5.60, 95% CI: 4.24-7.01), and zoledronic acid (WMD = 5.18, 95% CI: 4.62-5.74) showed the largest effect sizes. By 24 months (**Figure 4C**), treatment effects showed greater magnitude, with denosumab (WMD = 7.96, 95% CI: 5.38-10.52) and ibandronate (WMD = 7.65, 95% CI: 4.99-10.99) maintaining their leading positions.

#### 3.5.2 Total hip bone mineral density changes

At 12-month follow-up (**Figure 4B**, 15 studies, n=4,725), all interventions except risedronate showed significant superiority over the control group, with ibandronate (WMD = 4.23, 95% CI: 2.91-5.43) and denosumab (WMD = 3.87, 95% CI: 2.55-5.19) demonstrating the highest efficacy. By 24 months (**Figure 4D**), the ranking of intervention effectiveness changed, with eldecalcitol combined with risedronate demonstrating the largest effect size (WMD = 6.38, 95% CI: 3.38-9.61), followed by ibandronate (WMD = 5.64, 95% CI: 3.52-7.52) and denosumab (WMD = 5.31, 95% CI: 3.63-6.94).

#### 3.5.3 Intervention efficacy ranking

**Table 2** presents the SUCRA values ranking for each intervention at different follow-up time points and anatomical sites.

**Table 2:** Preventive efficacy ranking (SUCRA values) of interventions on BMD at lumbar spine and total hip sites.

Treatment	12 months		24 months	
	Lumbar spine	Total hip	Lumbar spine	Total hip
Zoledronic	0.72	0.60	0.68	0.55
Ibandronate	0.85	0.94	0.77	0.79
Risedronate	0.33	0.32	0.33	0.38
Denosumab	0.88	0.83	0.83	0.72
Eldecalcitol and Risedronate	0.54	0.62	0.67	0.89
Alendronate	0.11	0.14	0.22	0.14

BMD, bone mineral density; SUCRA, surface under cumulative ranking curve.

#### 3.5.4 12-month follow-up results

For lumbar spine bone mineral density changes, denosumab (SUCRA = 0.88) and ibandronate (SUCRA = 0.85) ranked highest, while alendronate (SUCRA = 0.11) ranked lowest. At the total hip site, ibandronate (SUCRA = 0.94) demonstrated the best efficacy, followed by denosumab (SUCRA = 0.83).

#### 3.5.5 24-month follow-up results

Treatment rankings changed over extended follow-up duration. For lumbar spine bone mineral density changes, denosumab (SUCRA = 0.83) maintained its leading position; however, at the total hip site, eldecalcitol combined with risedronate showed the highest SUCRA value (SUCRA = 0.89), which was superior to ibandronate (SUCRA = 0.79) and denosumab (SUCRA = 0.72). However, this analysis was based on only one included study (n=196), resulting in limited data stability and reliability, so this finding should be interpreted with caution.

#### 3.5.6 Pairwise comparison results

The league table from network meta-analysis demonstrated direct and indirect comparisons between all interventions (**Appendix 3**). At 12-month follow-up, the lumbar spine comparison between denosumab and ibandronate yielded a WMD of -0.02 (95% CI: -1.68, 1.70), while at the hip site, ibandronate showed a WMD of 0.36 (95% CI: -1.49, 2.10) compared to denosumab. At 24-month follow-up, the lumbar spine comparison between denosumab and ibandronate demonstrated a WMD of 0.32 (95% CI: -3.95, 3.78), whereas at the hip site, eldecalcitol combined with risedronate exhibited WMDs of 1.10 (95% CI: -2.25, 4.84) and 0.76 (95% CI: -2.72, 4.82) compared to denosumab and ibandronate, respectively, with all 95% confidence intervals encompassing zero, indicating no statistically significant differences between interventions.

### 3.6 Publication bias

**Figure 5:**
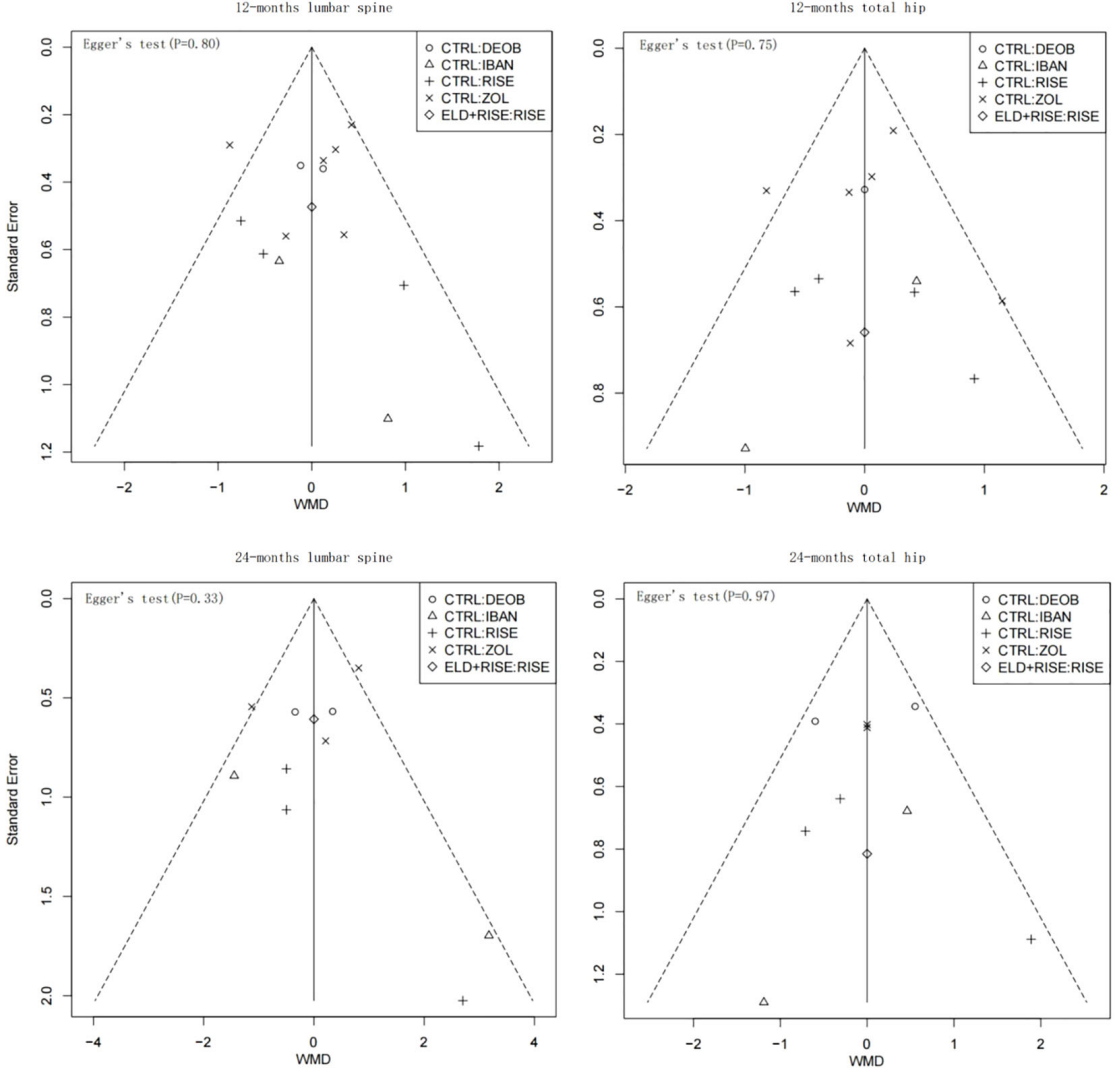
Funnel plots of different time points and anatomical sites. WMD, weighted mean difference.

Publication bias assessment for primary outcomes was conducted using funnel plots, with the horizontal and vertical axes representing weighted mean difference (WMD) and standard error, respectively. The WMD and its standard error displayed an inverted funnel-shaped distribution, which was utilized to detect potential small-study effects and selective reporting bias. As shown in **Figure 5**, included studies at different time points and anatomical sites were included in approximately symmetrically distributed groups on both sides of the X = 0 vertical line, indicating a low overall risk of publication bias. Notably, the 24-month total hip bone mineral density funnel plot exhibited the highest symmetry, with nearly all study points distributed within the funnel boundaries. It is worth noting that certain studies at the 24-month follow-up (such as CTRL/RISE and CTRL/IBAN) showed some deviation; however, this scatter pattern did not form systematic asymmetry, suggesting potential small-study effects, though not indicative of systematic publication bias. Egger’s regression test results indicated no statistical significance at any time point (P>0.05).

### 3.7 Model performance and evidence quality assessment

The Bayesian network meta-analysis demonstrated satisfactory model performance across multiple evaluation dimensions. Convergence assessment via visual inspection of Gelman diagnostic plots revealed that shrink factors for all parameters rapidly approached unity and remained stable throughout iterations, indicating adequate model convergence (**Appendix 4**), meanwhile, trace and density plots support the conclusion that the model fitting is reliable and the parameter estimates are valid (**Appendix 5**). Heterogeneity assessment showed low between-study heterogeneity across all outcomes (I² = 6-10%), suggesting minimal variability beyond sampling error (**Table 3**). Network consistency was evaluated through deviance information criterion (DIC) comparisons between consistency and inconsistency models: 12-month lumbar spine BMD (ΔDIC=0.21), 12-month total hip BMD (ΔDIC=0.00), 24-month lumbar spine BMD (ΔDIC=0.56), and 24-month total hip BMD (ΔDIC=0.20). All DIC differences were substantially below the threshold of 5, strongly supporting the consistency assumption (**Appendix 6**). Evidence quality assessment using the CINeMA framework demonstrated that most treatment comparisons at 12-month follow-up achieved high-quality evidence ratings, while some indirect comparisons at 24-month follow-up were downgraded to moderate confidence due to imprecision concerns (**Appendix 7**).

**Table 3:** Evaluation of heterogeneity and inconsistency.

Outcomes	Number of studies	DIC		Heterogeneity
		Consistency model	Inconsistency model	I2.cons
12months LS	16	60.17	60.38	6
12months Hip	15	56.98	56.98	7
24months LS	12	48.26	47.7	10
24months Hip	11	43.32	43.12	8

Through comprehensive meta-regression of all outcomes, we explored potential baseline characteristics that might contribute to heterogeneity among the included studies (**Table 4**). However, no significant sources of heterogeneity were identified, which is consistent with the inherently low heterogeneity observed in our study (I² < 10%). Sensitivity analyses excluding studies with high overall risk of bias confirmed the robustness of our findings (**Appendix 8**). Notably, in the 24-month lumbar spine and hip outcomes, after excluding two high-risk large-sample studies, the credible interval of zoledronic acid’s effect size widened significantly. This change is primarily attributed to the fact that the sample size of the excluded studies accounts for approximately 74% of the total sample size in this comparison. Although the point estimate has decreased, the direction of the positive effect remains stable.

**Table 4:** Network meta-regression.

Covariate	Shared beta (median and 95% CrI)			
	12months LS	12months Hip	24months LS	24months Hip
Publication year	0.36(-1.02, 1.62)	-0.04(-1.36, 1.45)	-1.15(-6.17, 3.14)	1.01(-2.10, 3.70)
Mean age	0.03(-1.25, 1.57)	0.27(-1.71, 1.30)	1.40(-2.15, 5.27)	0.03(-3.00, 2.04)
Baseline BMD	0.12(-1.36, 2.07)	-1.28(-3.66, 1.17)	1.12(-8.69, 10.73)	0.33(-5.31, 3.75)
Mean BMI	0.17(-0.94, 1.50)	-0.38(-1.61, 0.72)	0.30(-3.96, 5.61)	-0.89(-4.29, 2.02)
Co-intervention	-0.58(-1.45, 0.36)	-0.84(-1.61, 0.06)	-1.10(-3.94, 1.96)	-1.12(-3.02, 0.86)
Sample size	0.14(-0.93, 1.17)	0(-2.90, 2.67)	0.50(-2.63,4.00)	0.68(-1.93, 3.07)

BMI, body mass index; CrI, credible intervals; BMD,Bone Mineral Density; Co-intervention, Combined use of calcium supplements and vitamin D.

## 4 Discussion

In this research, we identified 23 qualifying randomized controlled trials that constructed a network with 6932 postmenopausal breast cancer patients. Most anti-resorptive agents within this network were not subjected to direct head-to-head trials, underscoring the value of our investigation. Due to the lack of horizontal comparisons among bone-protective agents in breast cancer patients receiving aromatase inhibitors therapy, determining optimal treatment strategies has become a challenge for clinicians. We compared the efficacy of six anti-resorptive interventions (denosumab, zoledronic acid, ibandronate, risedronate, alendronate, and eldecalcitol plus risedronate) for preventing bone loss at lumbar spine and hip sites across 12- and 24-month follow-up periods.

Our primary findings suggest that denosumab and ibandronate have solidified their positions as first-line bone-protective agents due to superior efficacy across anatomical sites. At 12-month follow-up, denosumab demonstrated the highest SUCRA value for lumbar spine bone mineral density improvement (SUCRA = 0.88, WMD = 5.63, 95% CI: 4.67-6.59), while ibandronate showed optimal efficacy for hip preservation (SUCRA = 0.94, WMD = 4.23, 95% CI: 2.91-5.43). Denosumab maintained its therapeutic advantage for lumbar spine preservation at 24 months (SUCRA = 0.83, WMD = 7.96, 95% CI: 5.38-10.52). At the 24-month endpoint, the combination therapy of eldecalcitol plus risedronate showed the highest SUCRA ranking for hip BMD (SUCRA = 0.89, weighted mean difference=6.38, 95% CI: 3.38-9.61), which was better than monotherapy. However, this result was derived from only one study with a small sample size (n=196), and the 95% confidence interval was relatively wide, indicating uncertainty in the estimate. Therefore, the combination regimen cannot be considered the definitive optimal choice for long-term hip protection, and more large-scale RCTs are needed to verify its efficacy.

The superior efficacy of denosumab can be attributed to its unique mechanism of action. As a monoclonal antibody targeting the receptor activator of nuclear factor-κB ligand (RANKL), denosumab completely blocks RANKL-RANK interaction on osteoclast precursors, thereby inhibiting osteoclast formation and function. Clinical evidence confirms denosumab’s significantly superior efficacy compared to bisphosphonates in treating lumbar spine osteoporosis (47). In the context of aromatase inhibitors therapy, this mechanism proves particularly crucial, as AI-induced estrogen deficiency upregulates RANKL expression (48). Consequently, denosumab effectively counteracts this pathway to reduce osteoclast activity, demonstrating excellent therapeutic effects against bone loss at both lumbar spine and hip sites. Notably, clinical practice has observed a rebound phenomenon following denosumab discontinuation(Brown JP,2013)(Tripto-Shkolnik L,2020), characterized by excessive activation of bone resorption(Lamy O,2025), marked elevation of bone turnover markers(Kendler DL,2010), and loss of previously gained BMD within 12–24 months(Watts NB,2024), which may increase the risk of vertebral fractures(Symonds C,2018). This rebound effect should be carefully considered in clinical decision-making, and appropriate follow-up or sequential interventions may be required after denosumab cessation to mitigate this risk.

Brown JP, Roux C, Törring O, Ho PR, Beck Jensen JE, Gilchrist N, Recknor C, Austin M, Wang A, Grauer A, Wagman RB. Discontinuation of denosumab and associated fracture incidence: analysis from the Fracture Reduction Evaluation of Denosumab in Osteoporosis Every 6 Months (FREEDOM) trial. J Bone Miner Res. 2013 Apr;28(4):746-52. doi: 10.1002/jbmr.1808.

Tripto-Shkolnik L, Fund N, Rouach V, Chodick G, Shalev V, Goldshtein I. Fracture incidence after denosumab discontinuation: Real-world data from a large healthcare provider. Bone. 2020 Jan;130:115150. doi: 10.1016/j.bone.2019.115150.

Lamy O, Everts-Graber J, Rodriguez EG. Denosumab for osteoporosis treatment: when, how, for whom, and for how long. A pragmatical approach. Aging Clin Exp Res. 2025 Mar 8;37(1):70. doi: 10.1007/s40520-025-02991-z.

Kendler DL, Roux C, Benhamou CL, Brown JP, Lillestol M, Siddhanti S, Man HS, San Martin J, Bone HG. Effects of denosumab on bone mineral density and bone turnover in postmenopausal women transitioning from alendronate therapy. J Bone Miner Res. 2010 Jan;25(1):72-81. doi: 10.1359/jbmr.090716.

Watts NB. What to Do When Denosumab Is Stopped? JAMA Netw Open. 2024 Nov 4;7(11):e2443879. doi: 10.1001/jamanetworkopen.2024.43879.

Symonds C, Kline G. Warning of an increased risk of vertebral fracture after stopping denosumab. CMAJ. 2018 Apr 23;190(16):E485-E486. doi: 10.1503/cmaj.180115.

Ibandronate, a third-generation nitrogen-containing bisphosphonate, demonstrates superior protective effects on cortical bone (particularly at the total hip site) due to its unique pharmacological properties and cortical bone physiology. With high bone affinity and moderate protein binding capacity, it rapidly distributes to highly vascularized cortical bone regions. Rogers’ study demonstrated that ibandronate achieves higher deposition concentrations in femoral cortical bone compared to vertebral bone (49). These properties explain ibandronate’s top-ranking SUCRA value for hip preservation in our analysis, making it particularly suitable for patients requiring prioritized cortical bone protection. As another third-generation nitrogen-containing bisphosphonate, zoledronic acid has often been more favored in previous guidelines and studies due to sufficient first-line research. However, in the ASCO-OH (CCO) Guideline Update (50), ibandronate, along with zoledronic acid, is listed as a first-line treatment option for adjuvant therapy in breast cancer patients.

Eldecalcitol, as an active vitamin D analog, exerts inhibitory effects on bone metabolism (51). Previous studies have demonstrated that prolonged bisphosphonate use may lead to diminished therapeutic efficacy over time (52). Notably, research by Mikio Kamimura et al. revealed that co-administration of eldecalcitol can significantly enhance the long-term effectiveness of bisphosphonate therapy (53). This synergistic effect explains the marked improvement in the SUCRA ranking of the eldecalcitol-plus-risedronate combination at the 24-month follow-up compared to the 12-month results in our study. While actively considering combination regimens involving active vitamin D analogs or other complementary agents may optimize therapeutic outcomes, this conclusion cannot be supported due to the limitations of the present study and requires further exploration through more first-line clinical studies.

Notably, bisphosphonates have demonstrated direct antitumor effects, capable of reducing tumor volume while simultaneously enhancing chemosensitivity (54). Previous meta-analyses have further confirmed that bisphosphonates significantly reduce breast cancer recurrence rates, distant metastasis rates, bone metastasis rates, and breast cancer-related mortality, particularly among postmenopausal women (55). This dual benefit of bone protection and potential anticancer effects adds to the therapeutic value of bisphosphonate therapy in breast cancer patients.

Previous systematic reviews and meta-analyses have evaluated bone-protective interventions in breast cancer patients receiving aromatase inhibitors therapy. The meta-analysis conducted by Bassatne & de Sire confirmed the bone-protective effects of both bisphosphonates and denosumab in postmenopausal breast cancer patients undergoing AI therapy (14, 15). However, while denosumab demonstrated therapeutic efficacy at both lumbar spine and hip sites, no clear hierarchical ranking was established among different bisphosphonate categories, nor were direct efficacy comparisons between these agents performed. Our findings are consistent with these studies regarding the overall efficacy of anti-resorptive agents but extend the evidence by providing comprehensive ranking through network meta-analysis. This discrepancy in ranking specificity may be attributed to the influences of indirect comparisons and the inclusion of combination therapies in our study.

Clinical practice guidelines and our network meta-analysis both highlight the importance of individualized bone-protective therapy selection. Our findings support a personalized treatment strategy based on individual risk profiles and treatment duration. For patients requiring rapid bone density improvement at lumbar spine, denosumab should be prioritized given its consistently superior SUCRA values across time points. In cases where hip bone preservation is the primary concern, ibandronate emerges as the optimal first-line choice at 12 months. For patients requiring extended therapy beyond 12 months, combination regimens incorporating eldecalcitol with risedronate may provide enhanced clinical benefits by concurrently targeting bone resorption inhibition and calcium metabolism regulation. This risk-stratified approach facilitates optimized treatment selection and improved clinical outcomes in breast cancer patients receiving aromatase inhibitors therapy. Additionally, consistent with clinical recommendations, adequate calcium (1000–1200 mg/d) and vitamin D (800–1000 IU/d) supplementation, lifestyle modifications (e.g., smoking cessation, limiting alcohol intake to ≤1 drink/day), and regular weight-bearing exercise (e.g., walking, stair climbing for 30 minutes 5 times/week) should be integrated as part of a comprehensive bone health management strategy for this patient population.

This study has several advantages compared to existing research. First, to our knowledge, this is the first network meta-analysis to comprehensively compare and rank all major anti-resorptive agents for bone protection in breast cancer patients receiving aromatase inhibitors therapy. This NMA provides valuable insight into clinical decision-making by establishing clear hierarchical rankings based on SUCRA values. Second, we analyzed therapeutic effects at multiple time points (12 and 24 months) and anatomical sites (lumbar spine and hip), providing more comprehensive evidence compared to existing pairwise meta-analyses. Furthermore, our Bayesian approach enabled robust indirect comparisons between interventions that have rarely been directly compared in head-to-head trials. Additionally, we evaluated the synergistic potential of combination therapy (eldecalcitol plus risedronate), which demonstrated superior long-term efficacy and represents an important finding for clinical practice.

However, our research also has certain limitations. First, the results may be affected by the limited number of studies for specific interventions, particularly denosumab and eldecalcitol combination therapy, which may lead to insufficient power for subgroup analyses and potential reporting bias. Second, all interventions were compared indirectly through a common control group, and indirect comparisons increase variance, which may lead to less precise effect estimates compared to direct head-to-head trials. Third, the follow-up duration was limited to a maximum of 24 months, restricting our ability to assess long-term treatment effects beyond this timeframe. Fourth, fracture risk evaluation, a critical clinical endpoint, was not assessed due to unavailable data in primary studies. In addition, adverse events such as osteonecrosis of the jaw, hypocalcemia, gastrointestinal toxicity, or atrial fibrillation were reported inconsistently across trials and were therefore not suitable for quantitative synthesis; the present network meta-analysis focuses on efficacy in terms of BMD changes only. Fifth, bisphosphonates were grouped by drug name rather than formulation (oral vs. intravenous), which may introduce potential heterogeneity due to differences in bioavailability and administration frequency between formulations. Future studies should explore the comparative efficacy of different formulations to optimize treatment selection. Finally, some studies may have been overlooked due to our focus on English-language publications, potentially introducing language bias.

## 5 Conclusion

In conclusion, this network meta-analysis demonstrates significant bone-protective benefits of anti-resorptive agents in postmenopausal breast cancer patients receiving aromatase inhibitor therapy. Although pairwise comparisons revealed no statistically significant differences between major interventions, ranking analyses suggest that denosumab is the preferred option for lumbar spine preservation and ibandronate is suitable for short-term hip bone protection. The combination of eldecalcitol and risedronate may be a promising option for long-term hip preservation; however, this conclusion is based only on a single small-sample study (n = 196) and should be interpreted with caution. These findings support personalized, anatomical site-specific treatment approaches, with clinical decision-making guided by individual patient factors, tolerability profiles, and risk-benefit considerations, particularly for combination therapies. Future head-to-head randomized controlled trials with extended follow-up are warranted to establish definitive treatment hierarchies and optimize therapeutic sequencing.

## Data Availability

The original contributions presented in the study are included in the article/**Supplementary Material**. Further inquiries can be directed to the corresponding author/s.
