# Supplementary material for: Comparative effectiveness of bone-protective interventions for aromatase inhibitors-induced bone loss in postmenopausal women with early breast cancer: a network meta-analysis
Source: Front Oncol. 2026 Jan 6;15:1638370. doi: 10.3389/fonc.2025.1638370 (PMC12815783; doi:10.3389/fonc.2025.1638370)
Supplement: Supplementary file 1 [file DataSheet1.docx]

**Catalogue**

[Appendix 1: Search strategy 5](#_Toc29046)

[Appendix 2: The risk of bias assessment for the individual included studies. 7](#_Toc8072)

[Figure 1 ROB2 figure (ITT) 7](#_Toc15843)

[Appendix 3 NMA results 9](#_Toc11914)

[Table 3.1: League Table (12 months LS) 9](#_Toc26911)

[Table 3.2: League Table (12 months Hip) 9](#_Toc15629)

[Table 3.3: League Table (24 months LS) 10](#_Toc12954)

[Table 3.4: League Table (24 months Hip) 10](#_Toc2163)

[Appendix 4: Diagnosis of convergence 11](#_Toc1240)

[Figure 4.1 Gelman plot (12 months LS) 11](#_Toc15687)

[Figure 4.2 Gelman plot (12 months Hip) 12](#_Toc25266)

[Figure 4.3 Gelman plot (24 months LS) 13](#_Toc11402)

[Figure 4.4 Gelman plot (24 months Hip) 14](#_Toc7194)

[Appendix 5:Trace and Density plot 15](#_Toc2961)

[Figure 5.1 Trace and Density plot(12 months LS) 15](#_Toc26394)

[Figure 5.2 Trace and Density plot(12 months Hip) 16](#_Toc29321)

[Figure 5.3 Trace and Density plot(24 months LS) 17](#_Toc11372)

[Figure 5.4 Trace and Density plot(24 months Hip) 18](#_Toc24246)

[Appendix 6：Model Performance Evaluation 19](#_Toc18877)

[Figure 6.1 Consistency and inconsistency model（12 months LS） 19](#_Toc7181)

[Figure 6.2 Consistency and inconsistency model（12 months Hip） 19](#_Toc4338)

[Figure 6.3 Consistency and inconsistency model（24 months LS） 20](#_Toc6632)

[Figure 6.4 Consistency and inconsistency model（24 months Hip） 20](#_Toc21229)

[Appendix 7：The result of CINeMA 21](#_Toc14403)

[Figure 7.1 CINeMA table（12 months LS） 21](#_Toc22540)

[Figure 7.2 CINeMA table（12 months Hip） 22](#_Toc21595)

[Figure 7.3 CINeMA table（24 months LS） 23](#_Toc21320)

[Figure 7.4 CINeMA table（24 months Hip） 24](#_Toc15747)

[Appendix 8：Sensitivity analyses 25](#_Toc29471)

[Figure 8.1 Exclude studies at overall high risk of bias （12 months LS） 25](#_Toc19791)

[Figure 8.2 Exclude studies at overall high risk of bias （12 months Hip） 26](#_Toc4621)

[Figure 8.3 Exclude studies at overall high risk of bias （24 months LS） 26](#_Toc18411)

[Figure 8.4 Exclude studies at overall high risk of bias （24 months Hip） 26](#_Toc14831)

#

# Appendix 1: Search strategy

Table 1.1 Search query and results for each database

| No. | Query | Results |
| --- | --- | --- |
| **Pubmed** | | |
| #1 | (bisphosphonate) or (alendronate) or (zoledronate) or (risedronate) or (ibandronate) or (minodronate) or (pamidronate) or (etidronate) or (clodronate) or (denosumab) or (elcatonin) or (salmon calcitonin) or (MHT) or (PTHa) or (teriparatide) or (abaloparatide) or (Vitamin D) or (alfacalcidol) or (calcitriol) or (eldecalcitol) or (menatetrenone) or (romosozumab) | 157248 |
| #2 | Aromatase Inhibitors"[Mesh] or (Inhibitors, Aromatase) or (Aromatase Inhibitor) or (Inhibitor, Aromatase) or (Anastrozole or Letrozole) or (Exemestane) | 17037 |
| #3 | "Breast Neoplasms"[Mesh] | 369581 |
| #4 | (Metabolic Bone Diseases) or (Bone Disease, Metabolic) or (Metabolic Bone Disease) or (Osteopenia) or (Osteopenias) or (Low Bone Density) or (Bone Density, Low) or (Low Bone Densities) or (Low Bone Mineral Density) | 122697 |
| #5 | (Postmenopause)[Mesh] OR (postmenopaus) OR (post menopaus) | 83044 |
| #6 | #1 AND #2 AND #3 AND #4 AND #5 | 235 |
| **Embase** | | |
| #1 | ('bisphosphonate':ti,ab OR 'alendronate':ti,ab OR 'zoledronate':ti,ab OR 'risedronate':ti,ab OR 'ibandronate':ti,ab OR 'minodronate':ti,ab OR 'pamidronate':ti,ab OR 'etidronate':ti,ab OR 'clodronate':ti,ab OR 'denosumab':ti,ab OR 'elcatonin':ti,ab OR 'salmon calcitonin':ti,ab OR 'MHT':ti,ab OR 'SERMs':ti,ab OR 'raloxifene':ti,ab OR 'bazedoxifene':ti,ab OR 'PTHa':ti,ab OR 'teriparatide':ti,ab OR 'abaloparatide':ti,ab OR 'vitamin D':ti,ab OR 'alfacalcidol':ti,ab OR 'calcitriol':ti,ab OR 'eldecalcitol':ti,ab OR 'menatetrenone':ti,ab OR 'romosozumab':ti,ab OR 'bone density conservation agent'/exp OR 'bisphosphonic acid derivative'/exp OR 'selective estrogen receptor modulator'/exp OR 'denosumab'/exp OR 'teriparatide'/exp) | 242236 |
| #2 | ('aromatase inhibitor'/exp OR 'anastrozole'/exp OR 'letrozole'/exp OR 'exemestane'/exp OR 'aromatase inhibitor':ti,ab OR 'anastrozole':ti,ab OR 'letrozole':ti,ab OR 'exemestane':ti,ab) | 45013 |
| #3 | ('breast cancer'/exp OR 'breast neoplas':ti,ab ) | 640933 |
| #4 | ( 'osteoporosis'/exp OR 'osteopenia'/exp OR 'bone density'/exp OR 'bone loss'/exp OR 'bone resorption'/exp OR 'bone metabolism disorder'/exp OR 'osteoporosis':ti,ab OR 'osteopenia':ti,ab OR 'bone loss':ti,ab OR 'low bone density':ti,ab OR 'low bone mineral density':ti,ab OR 'BMD':ti,ab OR 'bone resorption':ti,ab) | 354716 |
| #5 | ('postmenopause'/exp OR 'postmenopaus':ti,ab OR 'post menopaus':ti,ab) | 126395 |
| #6 | #1 AND #2 AND #3 AND #4 AND #5 | 970 |
| **Scopus** | | |
| #1 | TITLE-ABS-KEY(bisphosphonate OR alendronate OR zoledronate OR risedronate OR ibandronate OR minodronate OR pamidronate OR etidronate OR clodronate OR denosumab OR elcatonin OR "salmon calcitonin" OR MHT OR SERMs OR raloxifene OR bazedoxifene OR PTHa OR teriparatide OR abaloparatide OR "Vitamin D" OR alfacalcidol OR calcitriol OR eldecalcitol OR menatetrenone OR romosozumab) OR INDEXTERMS("bone density conservation agent" OR "bisphosphonate" OR "selective estrogen receptor modulator" OR "denosumab" OR "teriparatide") | 240116 |
| #2 | INDEXTERMS("aromatase inhibitor" OR "anastrozole" OR "letrozole" OR "exemestane") OR TITLE-ABS-KEY("aromatase inhibitor" OR anastrozole OR letrozole OR exemestane) | 33182 |
| #3 | INDEXTERMS("breast cancer" OR "breast neoplasm") OR TITLE-ABS-KEY("breast neoplas" OR "breast cancer") | 680523 |
| #4 | INDEXTERMS(osteoporosis OR "postmenopausal osteoporosis" OR osteopenia OR "bone density" OR "bone resorption") OR TITLE-ABS-KEY(osteoporosis OR osteopenia OR "bone loss" OR "low bone density" OR "low bone mineral density" OR BMD OR "bone resorption") | 307939 |
| #5 | INDEXTERMS(postmenopause) OR TITLE-ABS-KEY(postmenopaus OR "post menopaus") | 79633 |
| #6 | #1 AND #2 AND #3 AND #4 AND #5 | 827 |
| **Web of Science** | | |
| #1 | TS=(bisphosphonate OR alendronate OR zoledronate OR risedronate OR ibandronate OR minodronate OR pamidronate OR etidronate OR clodronate OR denosumab OR elcatonin OR "salmon calcitonin" OR MHT OR SERMs OR raloxifene OR bazedoxifene OR PTHa OR teriparatide OR abaloparatide OR "Vitamin D" OR alfacalcidol OR calcitriol OR eldecalcitol OR menatetrenone OR romosozumab) | 261042 |
| #2 | TS=("aromatase inhibitor" OR anastrozole OR letrozole OR exemestane) | 22637 |
| #3 | TS=("breast neoplas*" OR "breast cancer*") | 1096551 |
| #4 | TS=(osteoporosis OR osteopenia OR "bone loss" OR "low bone density" OR "low bone mineral density" OR BMD OR "bone resorption") | 356481 |
| #5 | TS=(postmenopaus* OR "post menopaus*") | 160895 |
| #6 | #1 AND #2 AND #3 AND #4 AND #5 | 506 |
| **Cochrane** | | |
| #1 | (bisphosphonate) or (alendronate) or (zoledronate) or (risedronate) or (ibandronate) or (minodronate) or (pamidronate) or (etidronate) or (clodronate) or (denosumab) or (elcatonin) or (salmon calcitonin) or (MHT) or (PTHa) or (teriparatide) or (abaloparatide) or (Vitamin D) or (alfacalcidol) or (calcitriol) or (eldecalcitol) or (menatetrenone) or (romosozumab) | 28048 |
| #2 | MeSH descriptor: [Breast Neoplasms] explode all trees | 20502 |
| #3 | MeSH descriptor: [Aromatase Inhibitors] explode all trees | 934 |
| #4 | MeSH descriptor: [Osteoporosis] explode all trees5489 | 5489 |
| #5 | #1 AND #2 AND #3 AND #4 | 17 |

# Appendix 2: The risk of bias assessment for the individual included studies.

Figure 1 ROB2 figure (ITT)


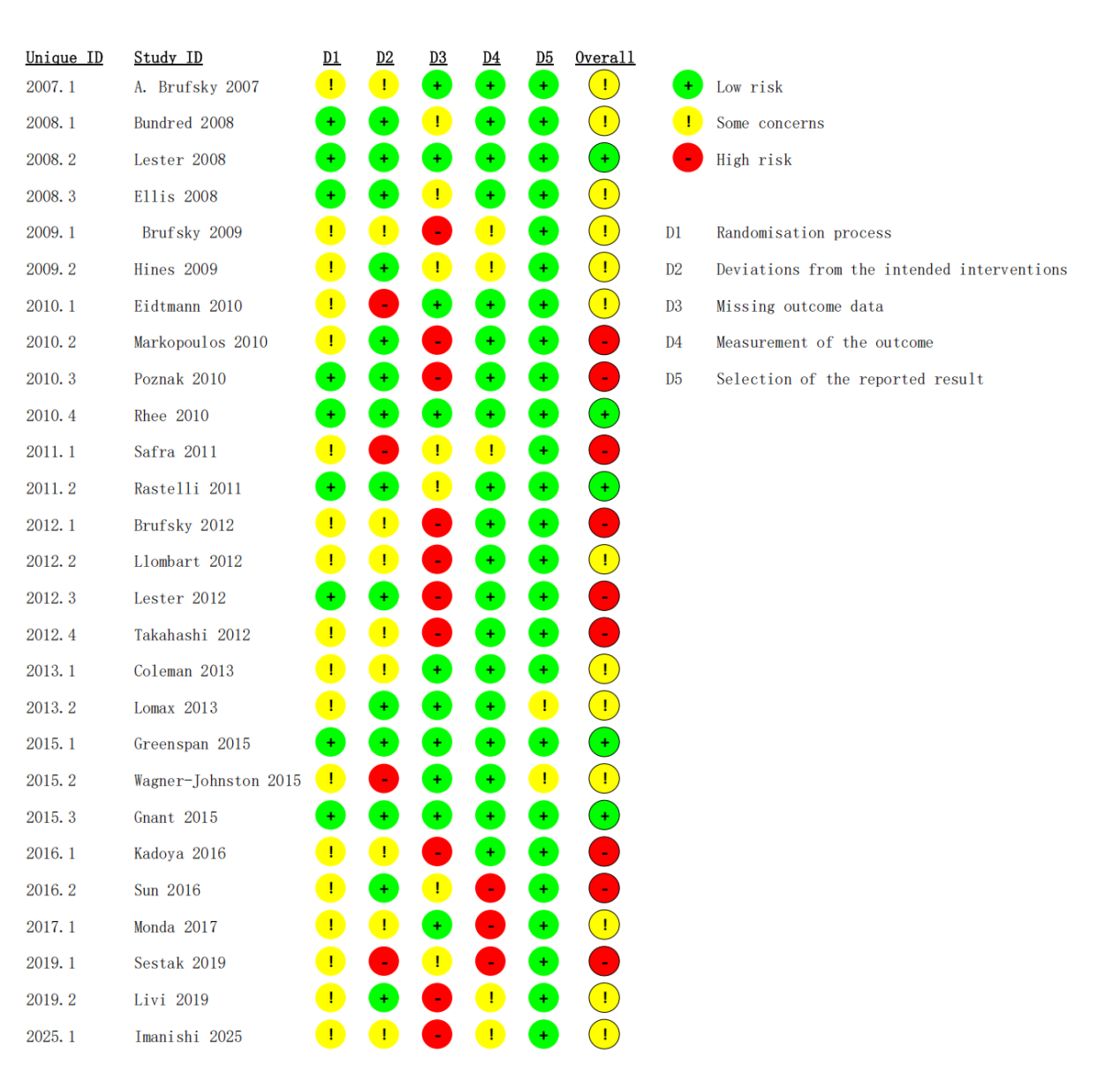


# Appendix 3 NMA results

Table 3.1: League Table (12 months LS)

#
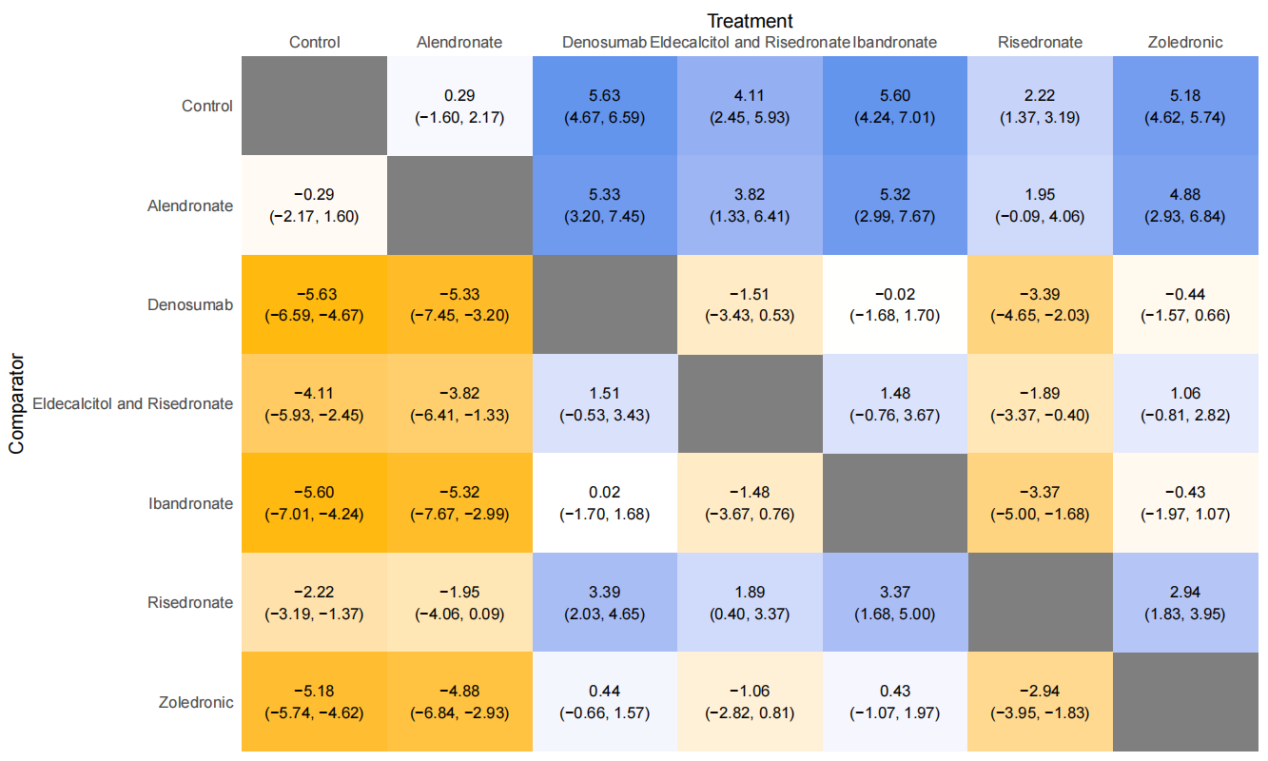


Table 3.2: League Table (12 months Hip)


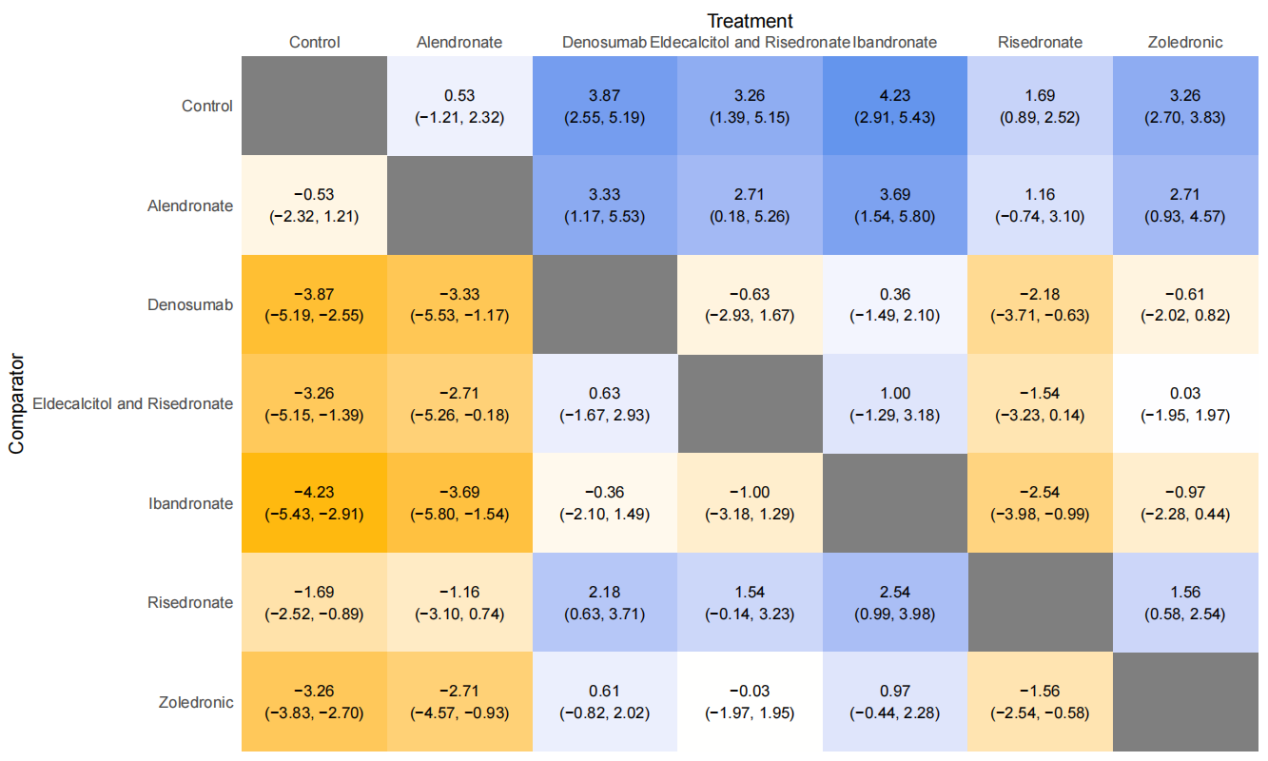


Table 3.3: League Table (24 months LS)

#
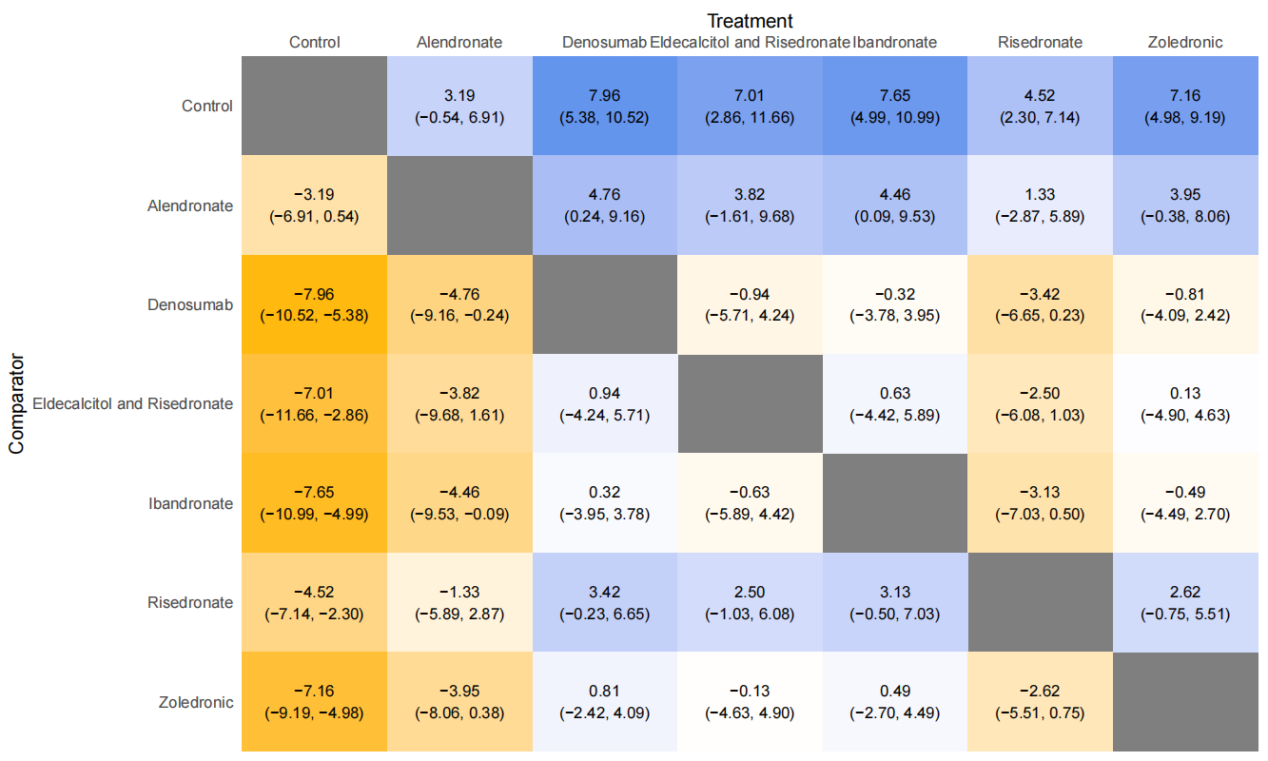


Table 3.4: League Table (24 months Hip)

#
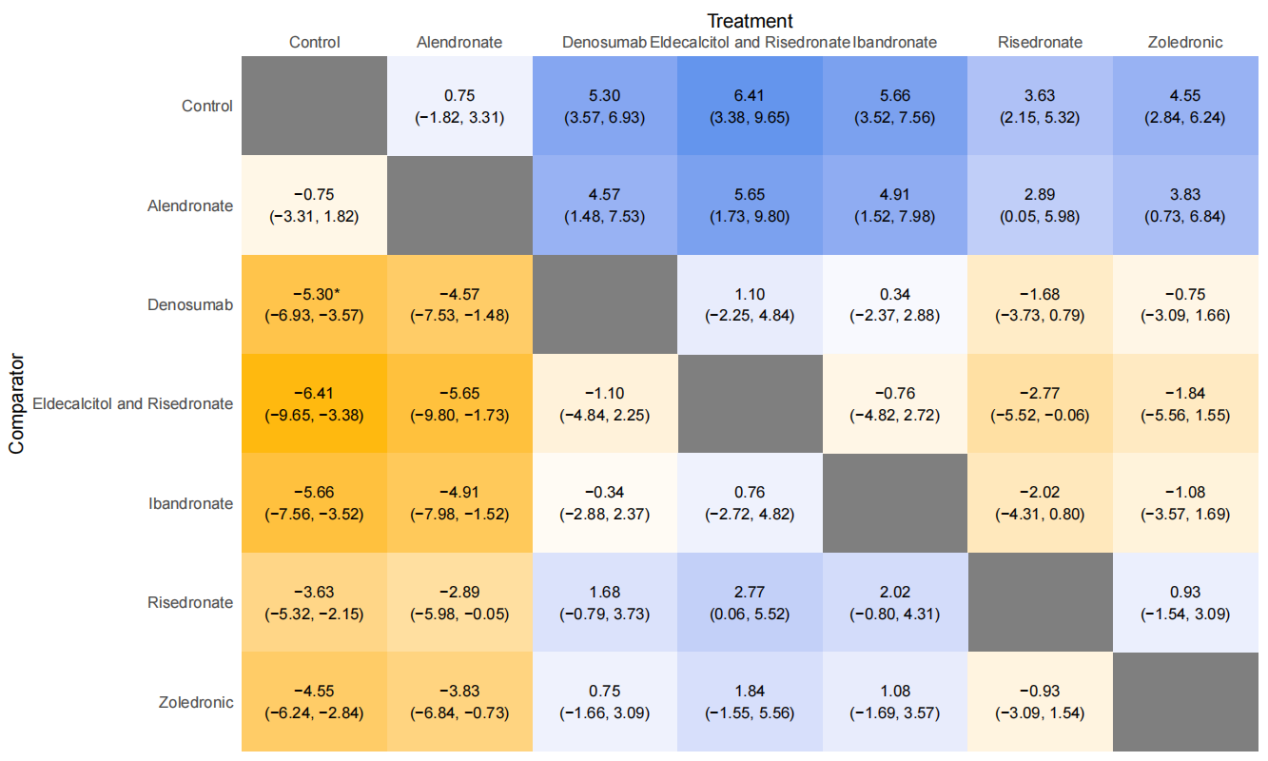


# Appendix 4: Diagnosis of convergence

Figure 4.1 Gelman plot (12 months LS)


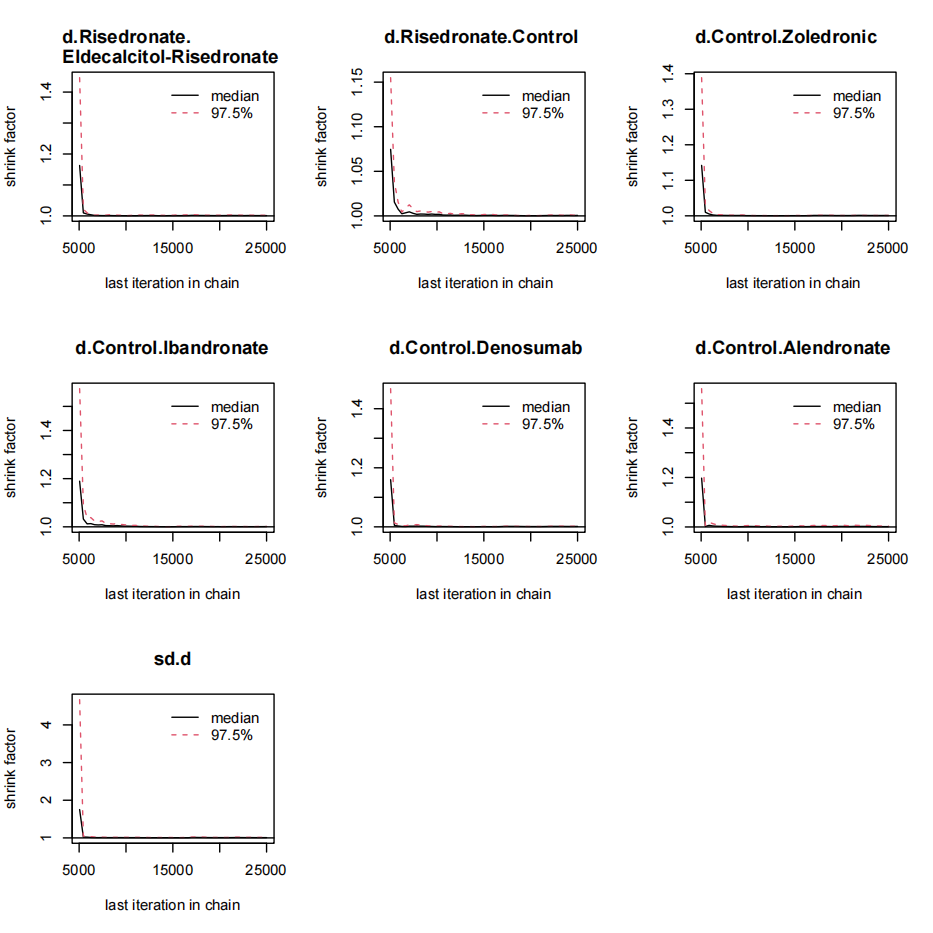


Figure 4.2 Gelman plot (12 months Hip)

**
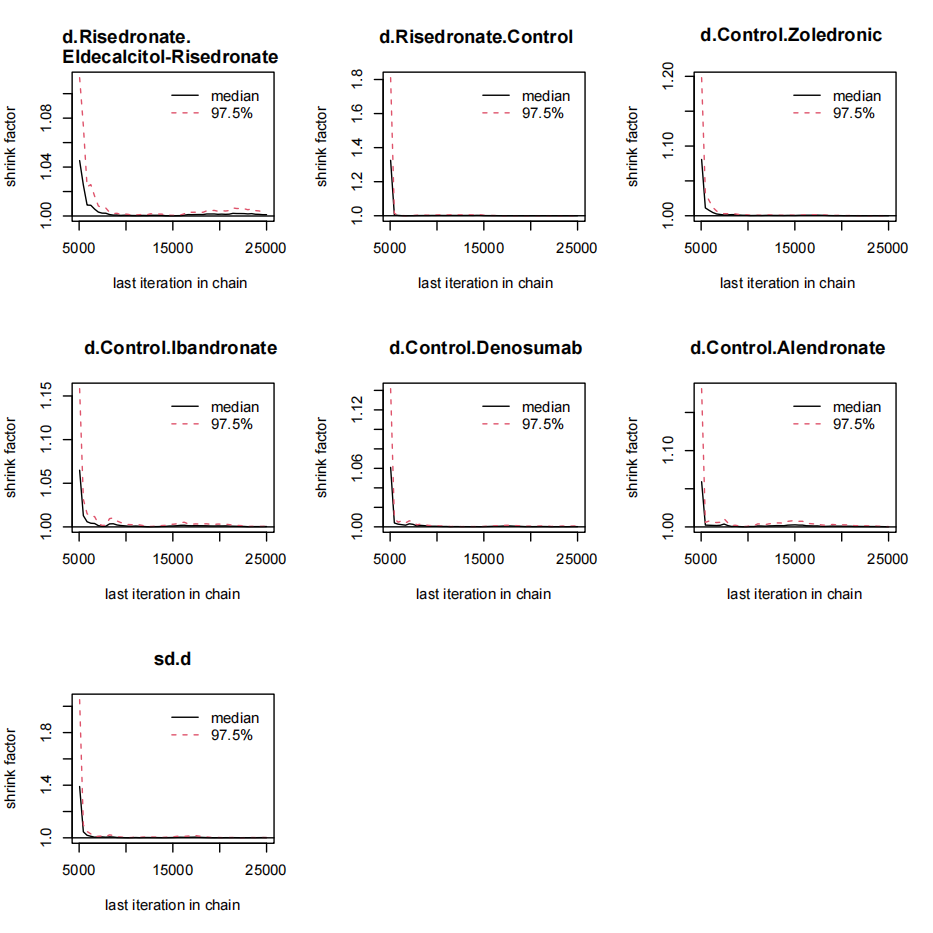
**

Figure 4.3 Gelman plot (24 months LS)

**
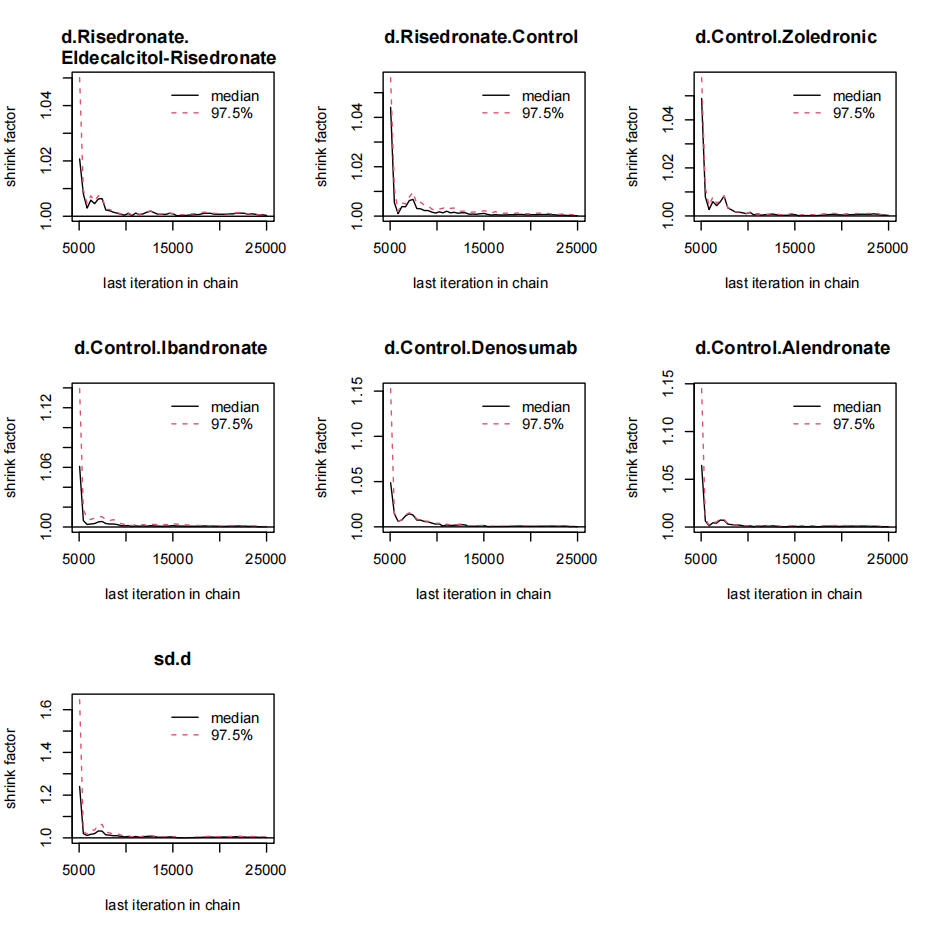
**

Figure 4.4 Gelman plot (24 months Hip)

**
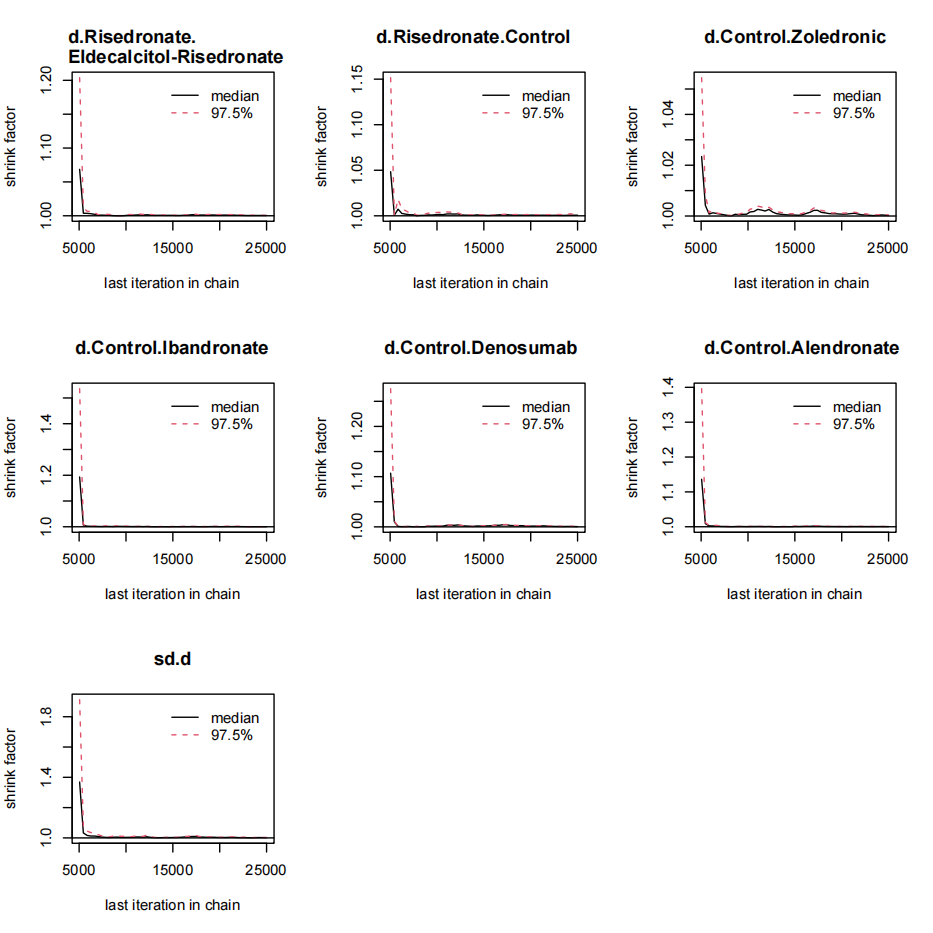
**

# Appendix 5:Trace and Density plot

Figure 5.1 Trace and Density plot(12 months LS)

**
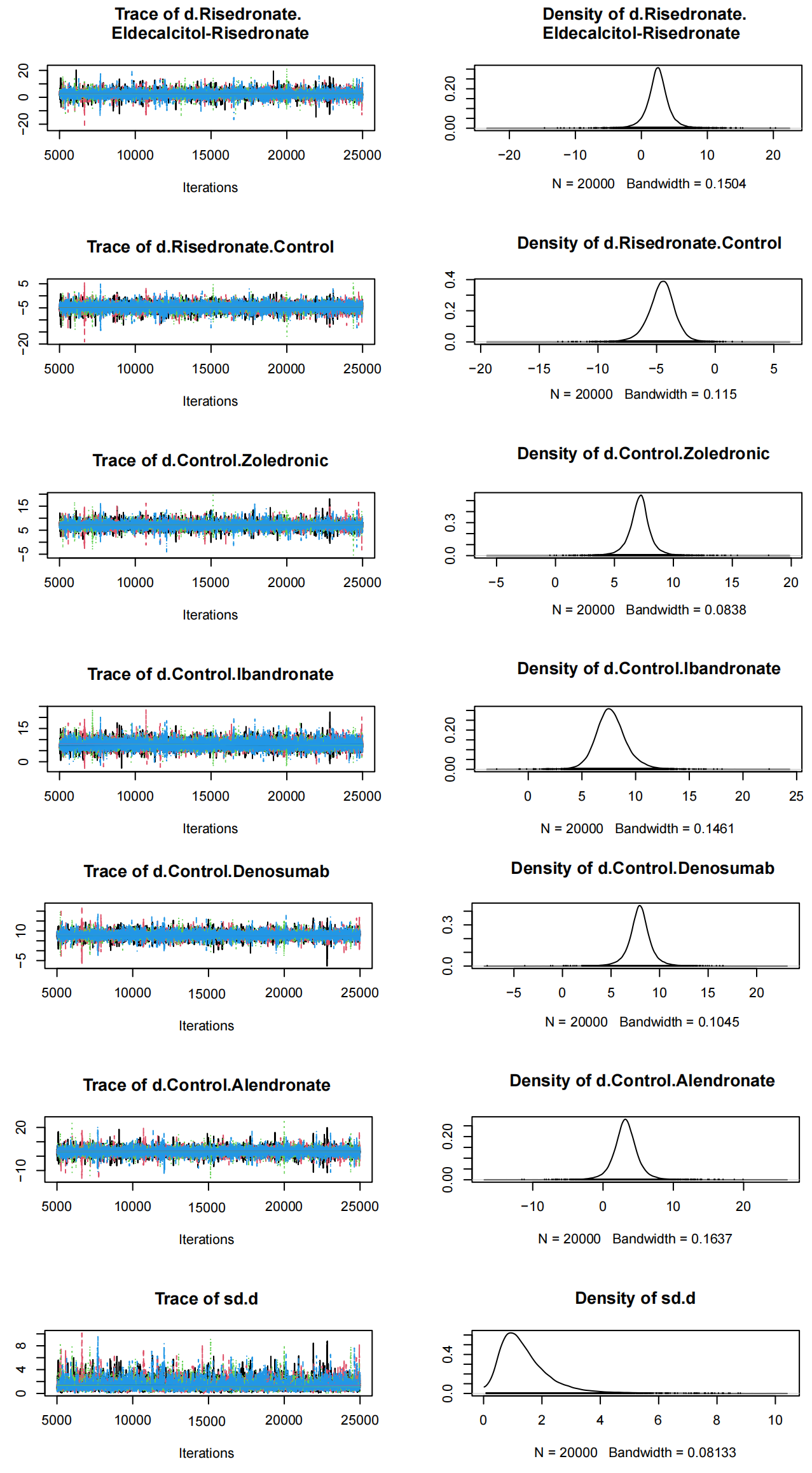
**

Figure 5.2 Trace and Density plot(12 months Hip)


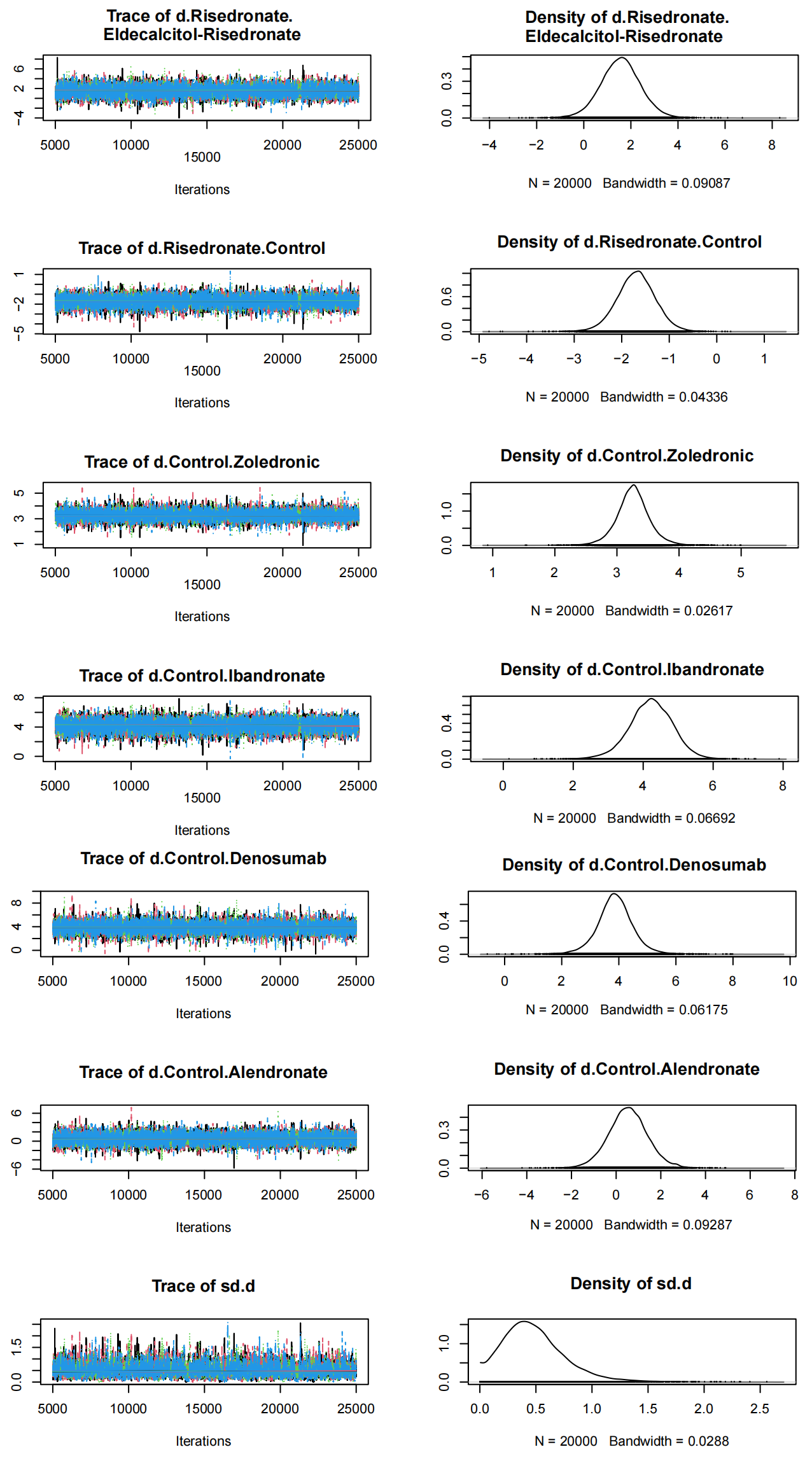


Figure 5.3 Trace and Density plot(24 months LS)


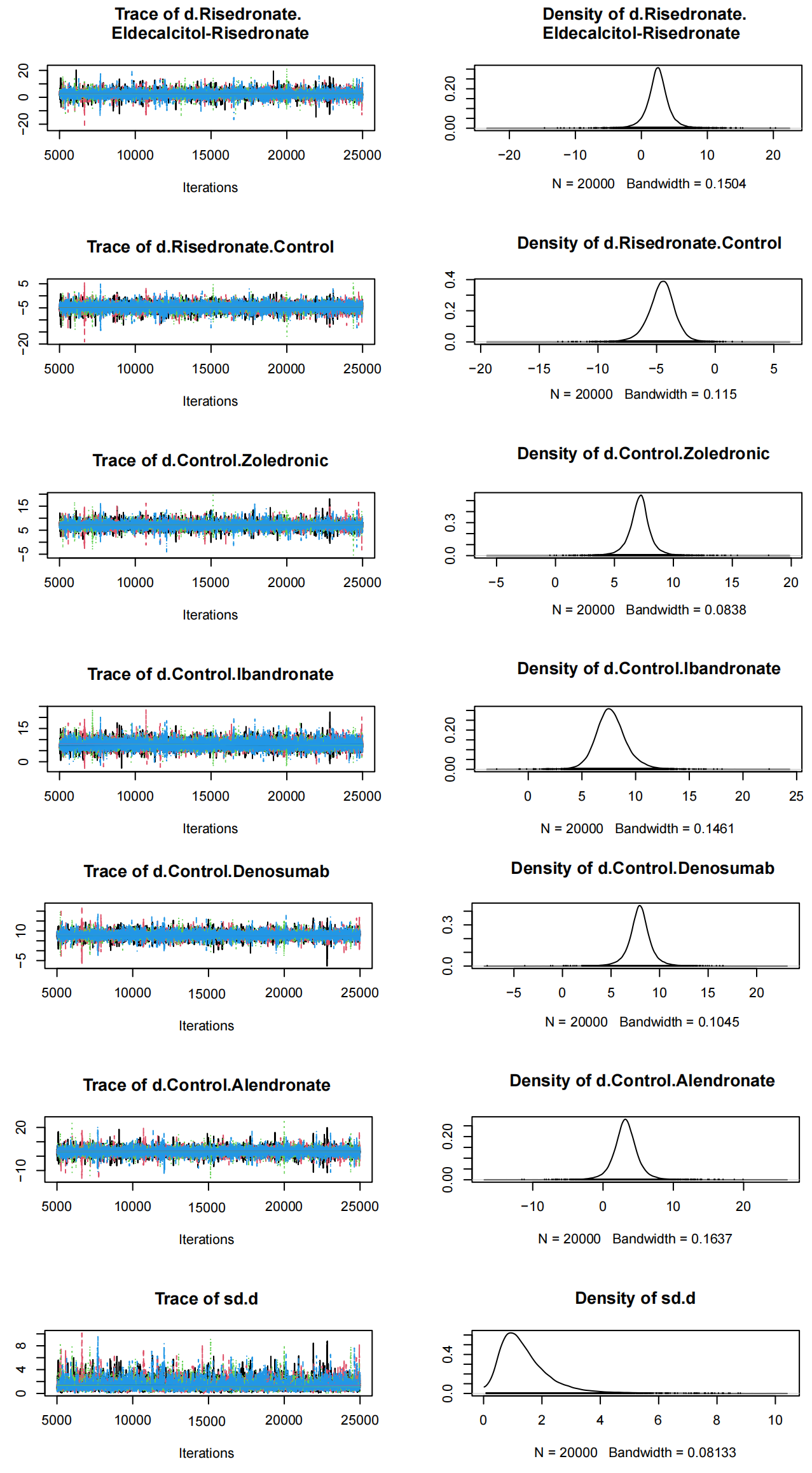


Figure 5.4 Trace and Density plot(24 months Hip)


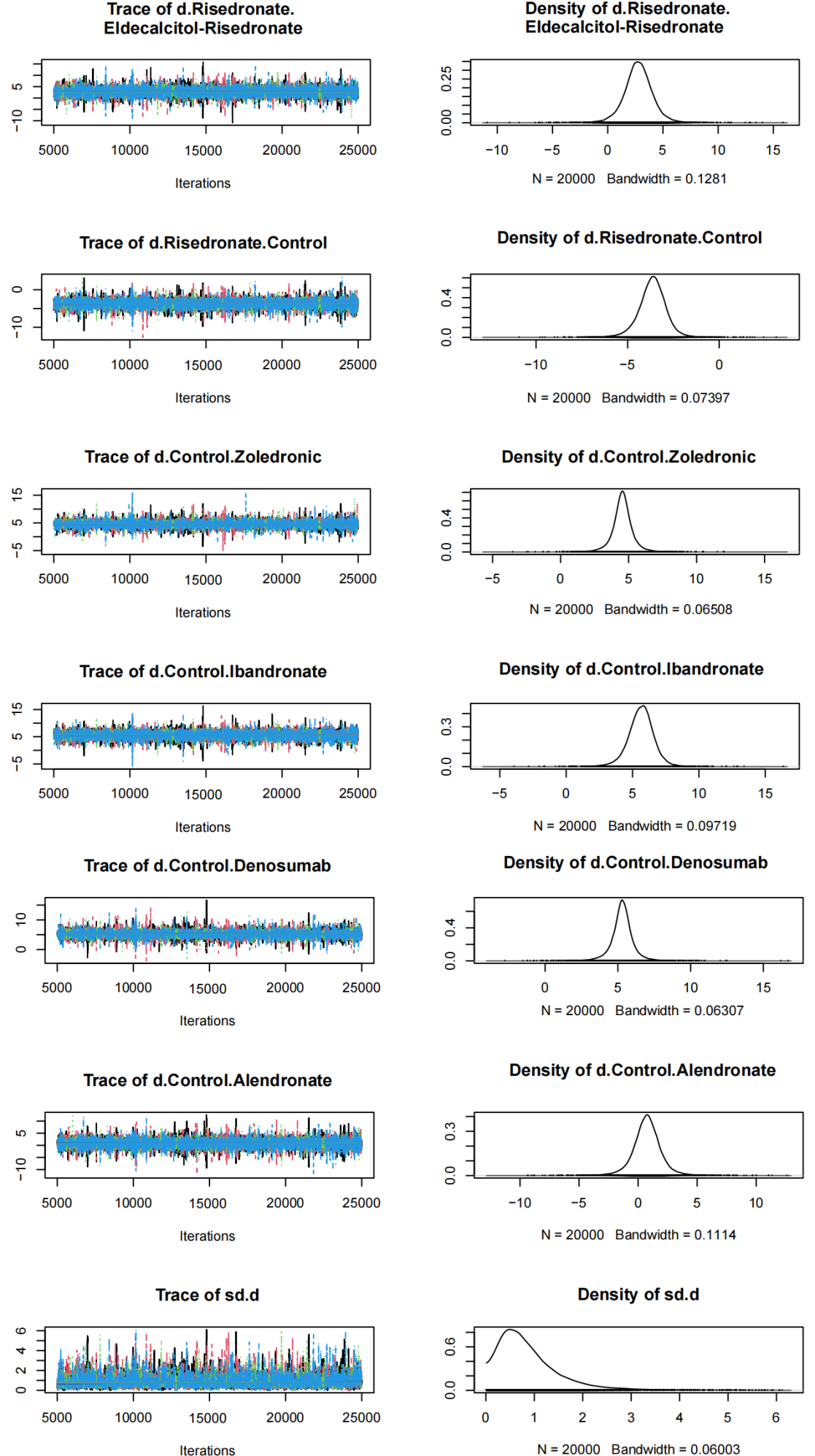


# Appendix 6：Model Performance Evaluation

Figure 6.1 Consistency and inconsistency model（12 months LS）

**
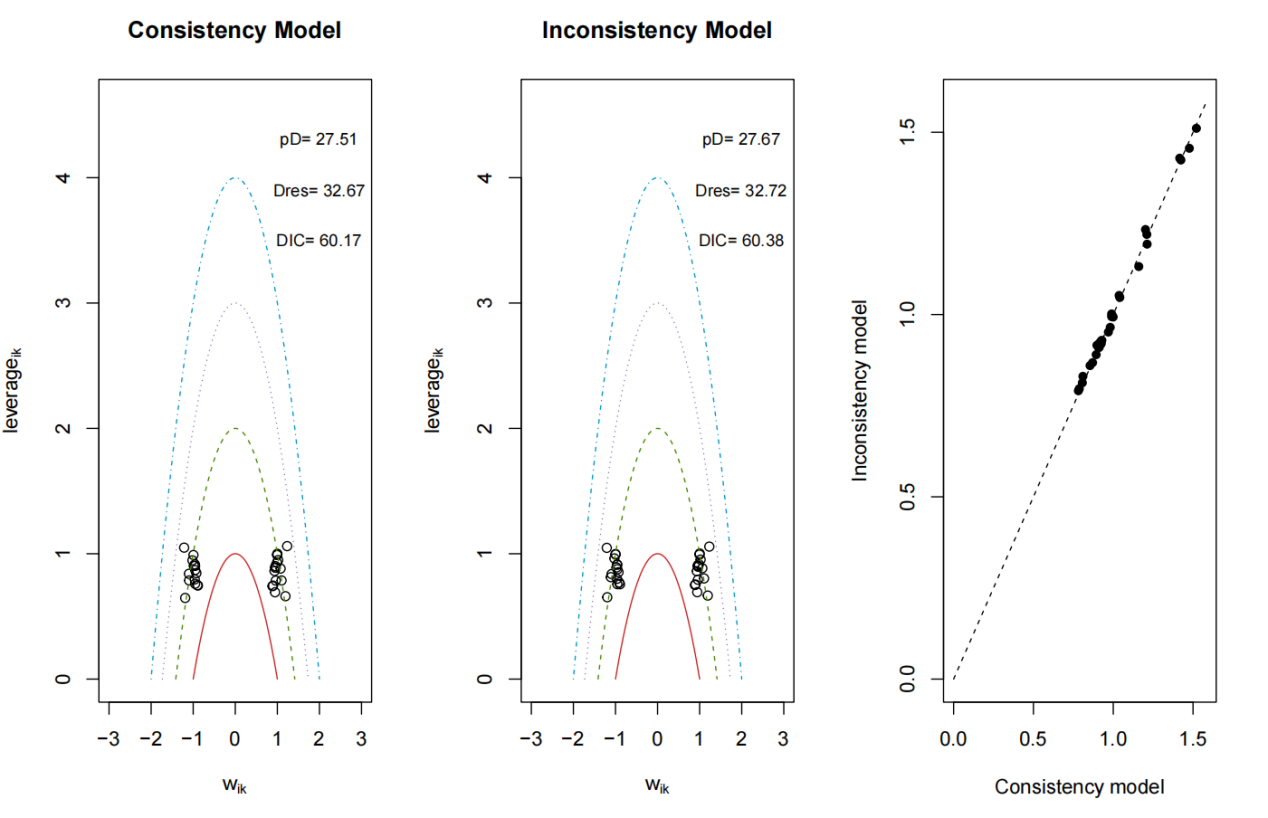
**

Figure 6.2 Consistency and inconsistency model（12 months Hip）

**
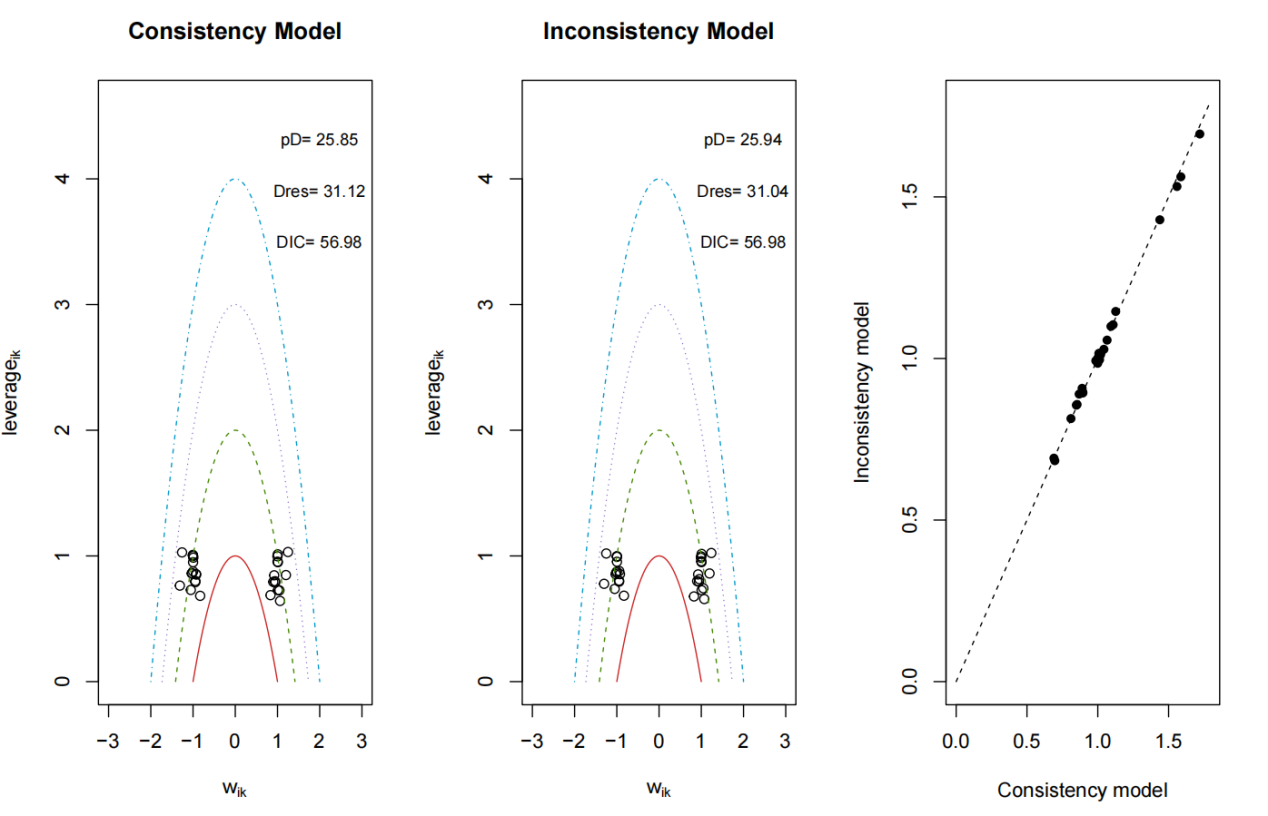
**

Figure 6.3 Consistency and inconsistency model（24 months LS）

**
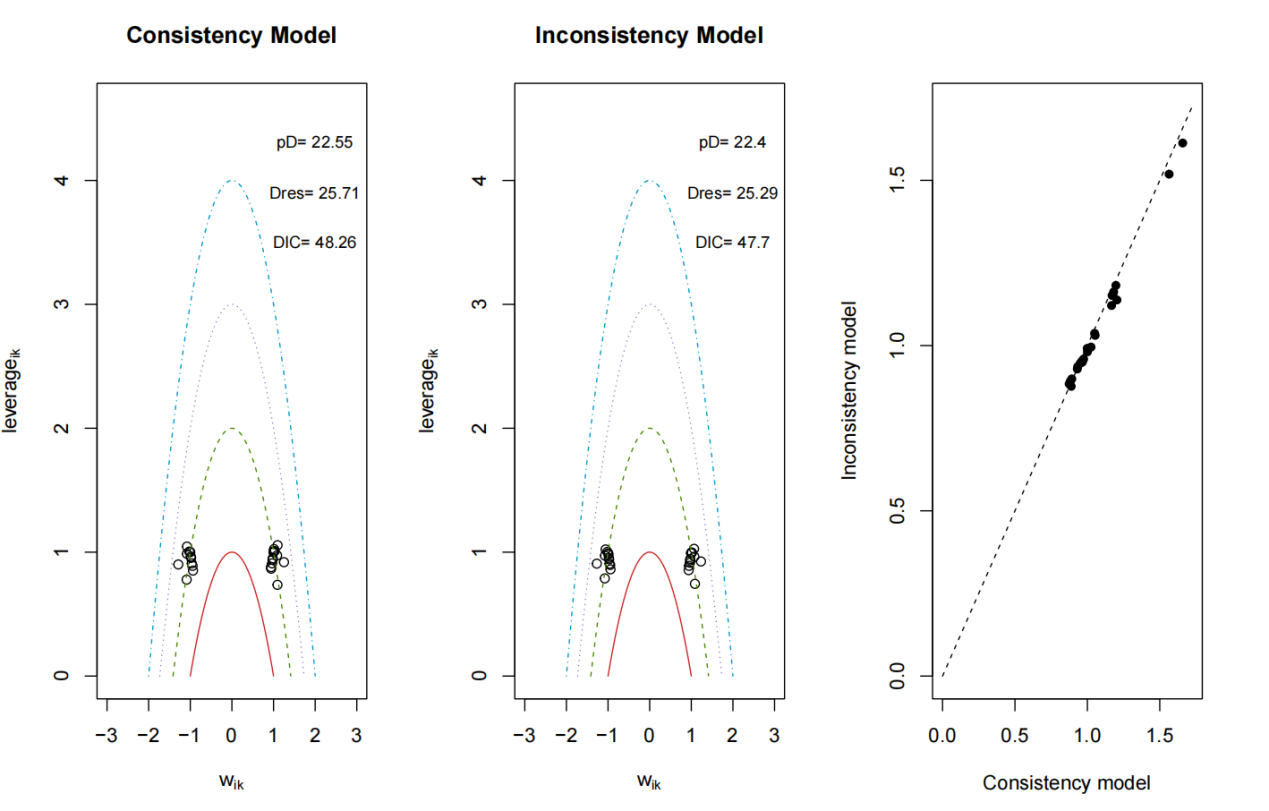
**

Figure 6.4 Consistency and inconsistency model（24 months Hip）


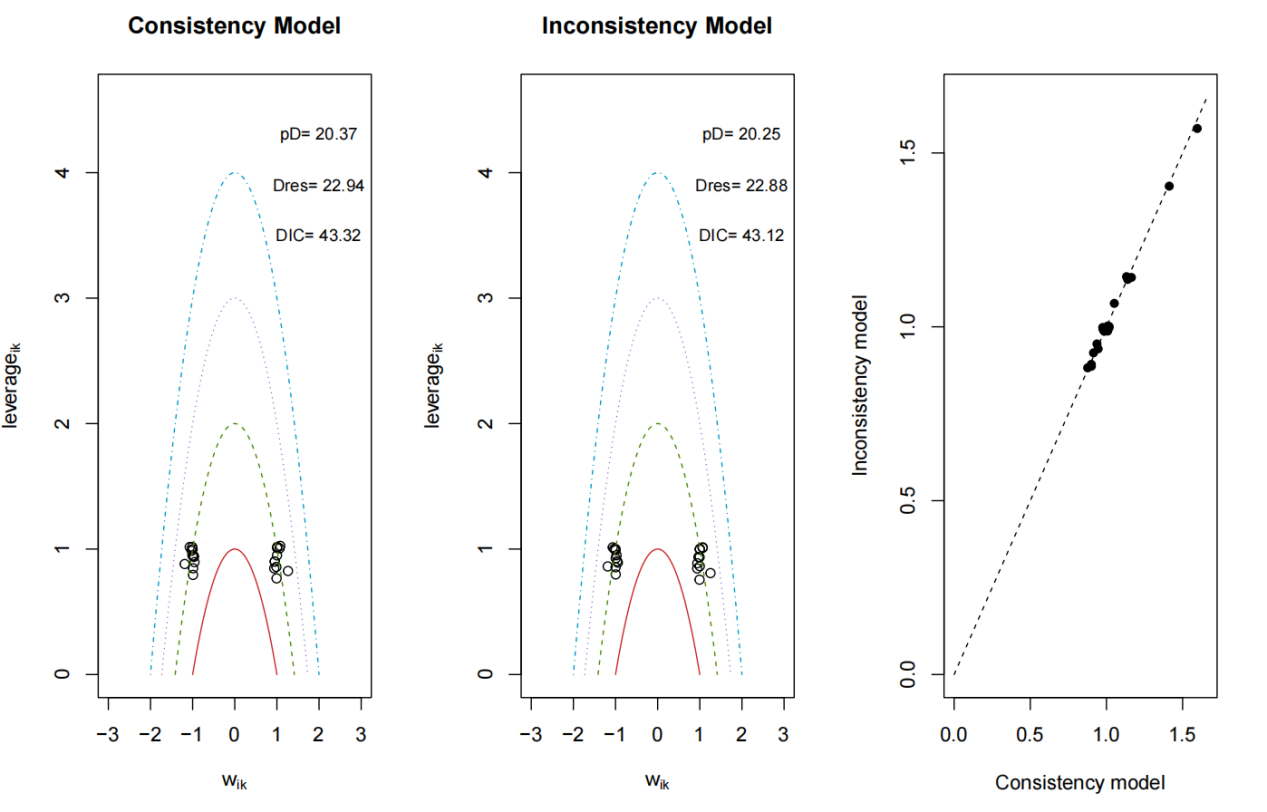


# Appendix 7：The result of CINeMA

Figure 7.1 CINeMA table（12 months LS）

Figure 7.2 CINeMA table（12 months Hip）

**
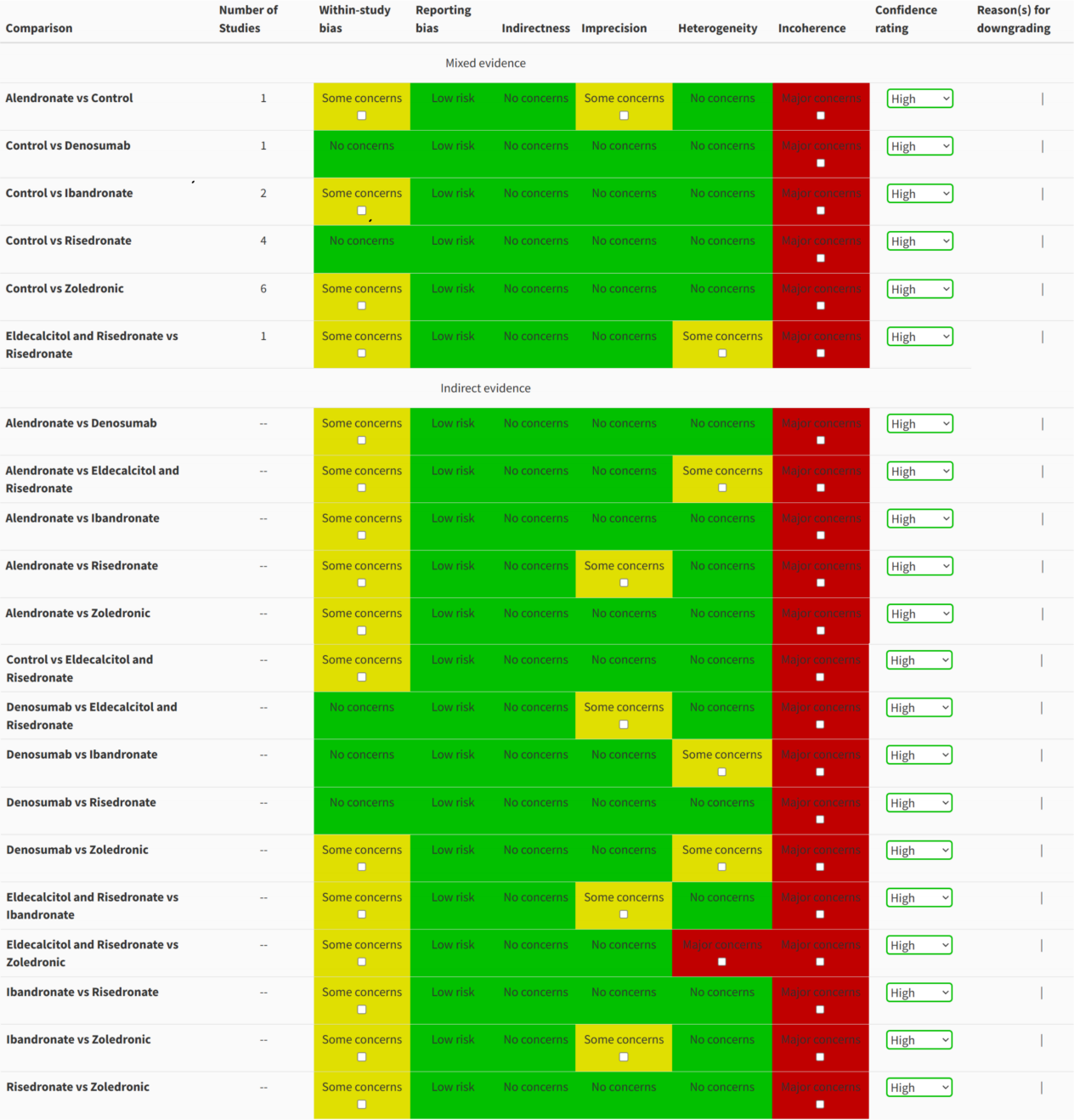
**

Figure 7.3 CINeMA table（24 months LS）

**
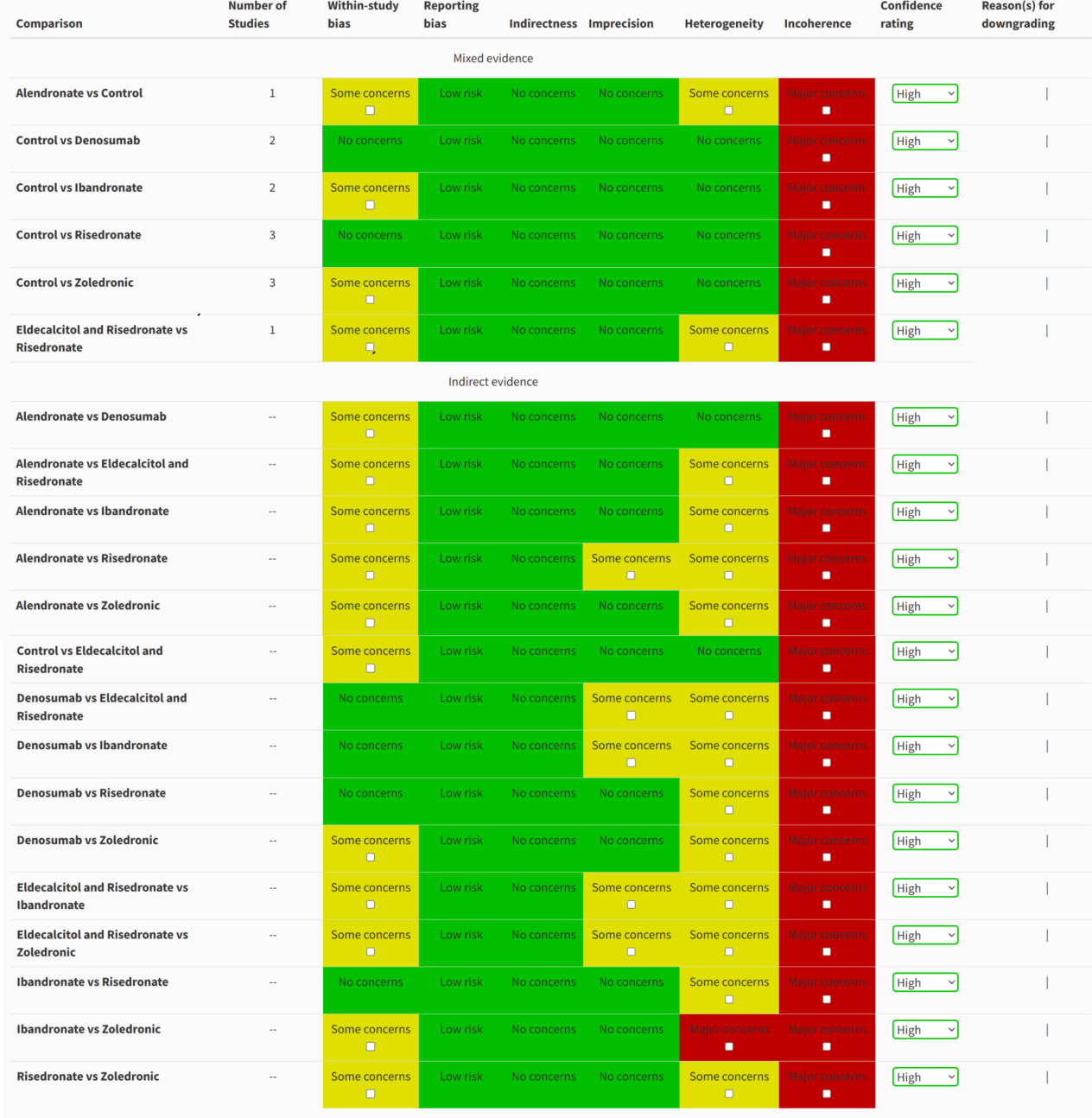
**

Figure 7.4 CINeMA table（24 months Hip）

**
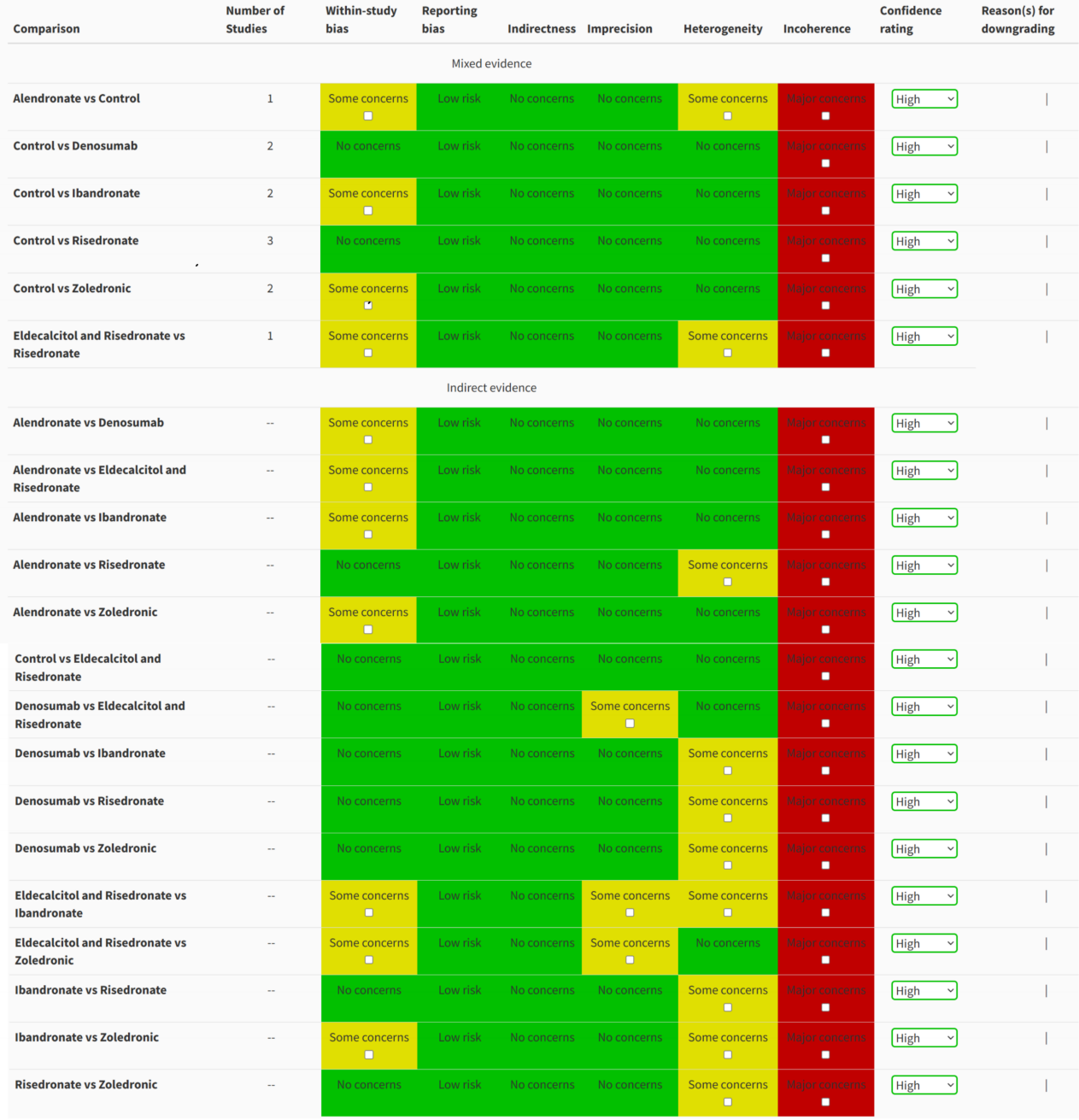
**

**Appendix 8：Sensitivity analyses**

We assessed the sensitivity of our findings by repeating each NMA after excluding studies at overall high risk of bias. Below we present the results from the changes in heterogeneity in each sensitivity analysis.

| **Including only studies with** | **Number of studies included** | **I²** | **DIC** |
| --- | --- | --- | --- |
| **12 months LS** | | | |
| All | 16 | 6% | 60.17 |
| studies at overall low to moderate risk of bias | 13 | 7% | 49.99 |
| **12 months Hip** | | | |
| All | 15 | 7% | 56.98 |
| studies at overall low to moderate risk of bias | 12 | 7% | 45.74 |
| **24 months LS** | | | |
| All | 12 | 10% | 48.26 |
| studies at overall low to moderate risk of bias | 10 | 11% | 40.39 |
| **24 months Hip** | | | |
| All | 11 | 8% | 43.32 |
| studies at overall low to moderate risk of bias | 10 | 7% | 39.55 |

Figure 8.1 Exclude studies at overall high risk of bias （12 months LS）

After excluding trials with high overall risk of bias, the hierarchy did not change significantly.

**
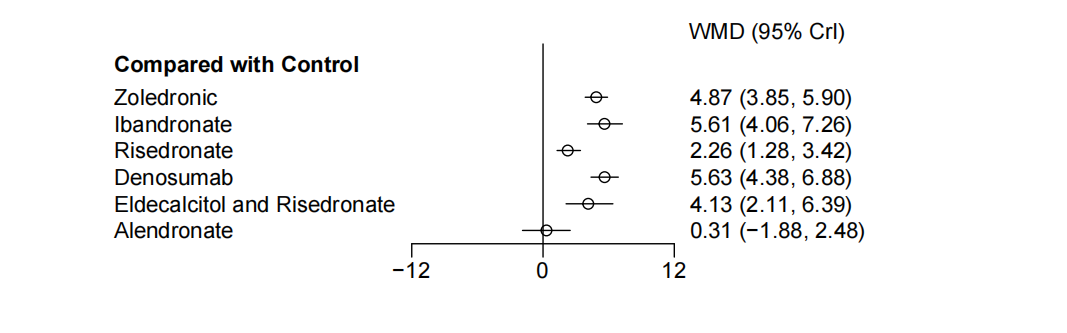
**

Figure 8.2 Exclude studies at overall high risk of bias （12 months Hip）

After excluding trials with high overall risk of bias, the hierarchy did not change significantly.

**
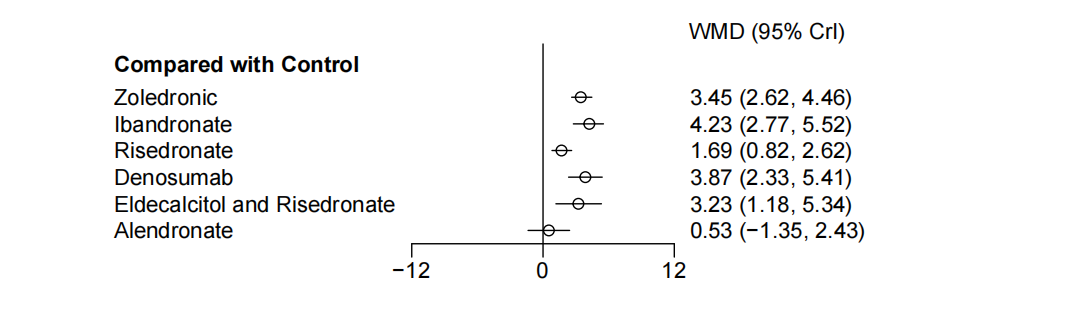
**

Figure 8.3 Exclude studies at overall high risk of bias （24 months LS）

After excluding trials with high overall risk of bias, the hierarchy did not change significantly. However, due to the exclusion of two large-sample studies (with a sample size accounting for approximately 74%) involving zoledronic acid, the confidence interval has widened significantly, while the overall direction of the effect remains unchanged.**
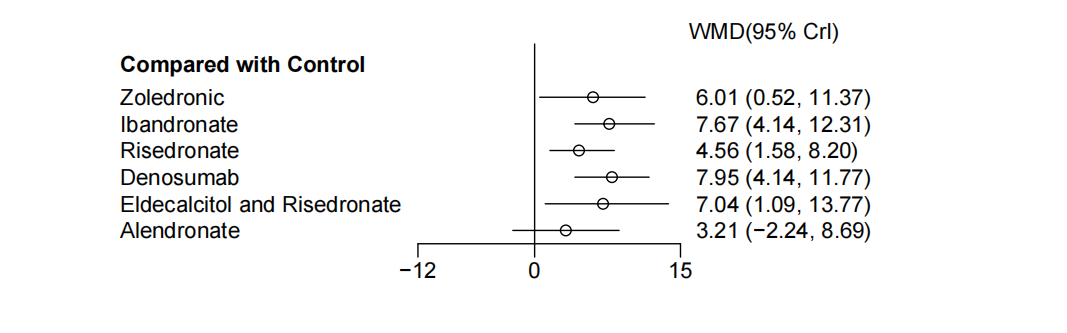
**

Figure 8.4 Exclude studies at overall high risk of bias （24 months Hip）

After excluding trials with high overall risk of bias, the hierarchy did not change significantly. However, due to the exclusion of two large-sample studies (with a sample size accounting for approximately 74%) involving zoledronic acid, the confidence interval has widened significantly, while the overall direction of the effect remains unchanged.

**
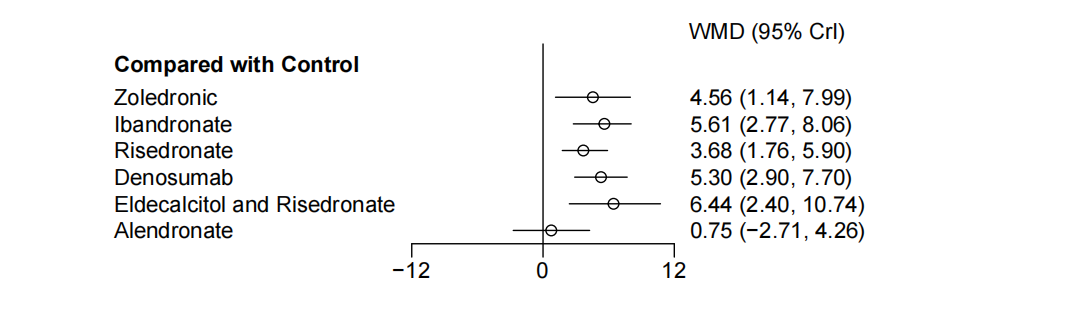
**
